# Insight into the Mechanistic Basis of the Hysteretic-Like Kinetic Behavior of Thioredoxin-Glutathione Reductase (TGR)

**DOI:** 10.1155/2018/3215462

**Published:** 2018-09-05

**Authors:** Juan L. Rendón, Mauricio Miranda-Leyva, Alberto Guevara-Flores, José de Jesús Martínez-González, Irene Patricia del Arenal, Oscar Flores-Herrera, Juan P. Pardo

**Affiliations:** Departamento de Bioquímica, Facultad de Medicina, Universidad Nacional Autónoma de México, Apartado Postal 70-159, 04510, D.F. México, Mexico

## Abstract

A kinetic study of thioredoxin-glutathione reductase (TGR) from* Taenia crassiceps* metacestode (cysticerci) was carried out. The results obtained from both initial velocity and product inhibition experiments suggest the enzyme follows a two-site ping-pong bi bi kinetic mechanism, in which both substrates and products are bound in rapid equilibrium fashion. The substrate GSSG exerts inhibition at moderate or high concentrations, which is concomitant with the observation of hysteretic-like progress curves. The effect of NADPH on the apparent hysteretic behavior of TGR was also studied. At low concentrations of NADPH in the presence of moderate concentrations of GSSG, atypical time progress curves were observed, consisting of an initial burst-like stage, followed by a lag whose amplitude and duration depended on the concentration of both NADPH and GSSG. Based on all the kinetic and structural evidence available on TGR, a mechanism-based model was developed. The model assumes a noncompetitive mode of inhibition by GSSG in which the disulfide behaves as an affinity label-like reagent through its binding and reduction at an alternative site, leading the enzyme into an inactive state. The critical points of the model are the persistence of residual GSSG reductase activity in the inhibited GSSG-enzyme complexes and the regeneration of the active form of the enzyme by GSH. Hence, the hysteretic-like progress curves of GSSG reduction by TGR are the result of a continuous competition between GSH and GSSG for driving the enzyme into active or inactive states, respectively. By using an arbitrary but consistent set of rate constants, the experimental full progress curves were successfully reproduced* in silico*.

## 1. Introduction

Thioredoxin-glutathione reductase (TGR E.C. 1.8.1.B1) represents an interesting splicing variant of the animal thioredoxin reductase (TR), featured by the presence of a glutaredoxin-like domain appended at the N-terminal end of the TrxR module [1, 2]. As a member of the disulfide reductase family, TGR is a NADPH-dependent homodimeric flavoenzyme with a dithiol/disulfide redox center located at the* si* face of the FAD prosthetic group [2]. As in mammalian TR [3], a selenocysteine residue located at the C-terminal end of the enzyme is also catalytically essential [4]. Unlike typical TR, however, TGR is also able to reduce oxidized glutathione (GSSG) at significant rates and to perform thiol/disulfide exchanges [1, 4], thus making it a multifunctional enzyme. Such additional catalytic capabilities are dependent on the presence of the Grx-like domain. Both the Grx-like domain and the C-terminal redox center of the enzyme are essential in the reduction of GSSG, as derived from site-directed mutagenesis studies [4–7]. Interestingly, the two redox centers are far away from one another as revealed by the three-dimensional structure of TGR from the blood fluke* Schistosoma mansoni* [8, 9], suggesting that TGR acts as a two-site enzyme during GSSG reduction. It has been proposed that, during the catalytic cycle of TGR with GSSG as the substrate, electrons must be shuttled from the reduced selenol/thiol couple toward the redox center of the Grx-like domain [9, 10], involving a large conformational transition of the C-terminal arm of the neighbor subunit.

The presence of the enzyme has been demonstrated in animals [1, 4], human [11], as well as in the parasitic representatives of the flatworms [12–17]. The latter organisms lack the typical glutathione reductase (GR) and TR enzymes, and TGR is the only disulfide reductase involved in the regeneration of the reduced state of both thioredoxin (trx) and glutathione. Hence, it has been considered as a potential drug target for an antihelminthic therapy [18–20]. However, in spite of its unusual tertiary structure and multifunctional nature, no detailed kinetic study of TGR has been carried out. In a previous work the purification and general kinetic properties of TGR from the larval stage (cysticerci) of* Taenia crassiceps* were reported [14]. Particularly noticeable was the existence of an atypical kinetic behavior with GSSG as the substrate, characterized by a lag time in the time courses at moderate or high concentrations of the disulfide. The magnitude of the lag time depended on enzyme concentration and of the presence of disulfide reducing reagents [14]. Such atypical kinetics was considered as a kind of hysteresis [21]. The same kinetic phenomenon was reported in TGR from larval* Echinococcus granulosus* [5] and* Taenia solium* cysticerci [17] and in the enzyme from the adult stage of the flukes* Fasciola hepatica* [15],* Fasciola gigantica* [16], and* Schistosoma mansoni* [7]. Thus, the GSSG-dependent atypical kinetics appear to be a common feature in TGR. Even in the recombinant enzyme from human such atypical kinetic behavior was observed [11]. However, no detailed molecular mechanism for such phenomenon is yet available. It was proposed that the atypical time courses of TGR were dependent on the covalent modification through glutathionylation of two structural cysteine residues of the enzyme [5]. Such model, however, shows serious faults as was demonstrated [15]. On the other hand, the potential role that the substrate NADPH could play in the atypical kinetics of TGR has not been investigated. In all the works reporting the apparent hysteretic behavior of the enzyme, NADPH concentrations of 100 *μ*M or higher have been used [5, 7, 11, 14–17]. In order to elucidate the molecular basis of the hysteretic behavior of the enzyme, in the present work, the kinetic mechanism of wild type TGR from* T. crassiceps* was investigated. Furthermore, the effect of NADPH on the atypical kinetic behavior of TGR was also studied. Based on both the kinetic and the crystallographic evidence on TGR, a comprehensive model consistent with all the experimental observations is put forward and tested through* in silico* simulations. All the experimental observations regarding the kinetic behavior of the enzyme were successfully reproduced by the model.

## 2. Materials and Methods

### 2.1. Reagents

2′5′ ADP–Sepharose 4B was obtained from Amersham Pharmacia Biotech (Uppsala, Sweden). All others chemicals were obtained from Sigma Chemical Company (St. Louis, MO, USA) and used without further purification. Water purified by reverse osmosis was used in the preparation of solutions.

### 2.2. Growth of* T. crassiceps* Cysticerci

The HYG strain of* T. crassiceps* was used as a source of TGR. The cysticerci growth conditions, as well as its extraction, rupture, and preparation of a crude homogenate have been described elsewhere [22].

### 2.3. Enzyme

TGR from the cytosolic fraction of larval* T. crassiceps* was purified to homogeneity as previously described [14]. Enzyme solutions were stored at -20°C until use.

### 2.4. Enzyme Assays

The disulfide reductase activity assays of TGR were carried out in an Agilent 8453 uv/visible spectrophotometer (Hewlett Packard) fitted with a thermostated cell holder. All the kinetic experiments were performed at 25°C in 0.1 M Tris/HCl buffer (pH 7.8) containing 1 mM EDTA (buffer A) in a final volume of 1 mL.

The GSSG reductase activity of TGR was determined by following the decrease in absorbance at 340 nm as a consequence of NADPH oxidation [23]. Both NADPH and GSSG were incubated in buffer A during two min in order to obtain the baseline. Then, the reaction was started by adding a small enzyme aliquot. An extinction coefficient at 340 nm of 6220 M^−1^ cm^−1^ for NADPH was used in the calculations of initial velocities. In those experiments dealing with the effect of GSSG on the reduction of oxidized thioredoxin (Trx), human Trx was used as substrate. In this case, the reaction mixture contained 100 *μ*M NADPH, human Trx at the corresponding concentration, and 2 mM GSSG at a final volume of 120 *μ*L in buffer A. The reaction was started by adding a small TGR aliquot.

The concentration of both NADPH and NADP^+^ was determined spectrophotometrically by reading the absorbance of an aliquot of the corresponding stock solution at either 340 nm (*ε* = 6220 M^−1^ cm^−1^) or 259 nm (*ε* = 18000 M^−1^ cm^−1^) for NADPH or NADP^+^, respectively. As regards GSSG, its concentration in the stock solution was calculated through enzyme assays by mixing an aliquot of the disulfide with TGR in the presence of an excess of NADPH. After exhaustion of GSSG, its concentration was determined from the total change in absorbance at 340 nm.

### 2.5. Protein Determination

The monomer concentration in the stock solutions of TGR was determined from its absorbance at 462 nm using a molar extinction coefficient of 11.3 mM^−1^ cm^−1^ for protein bound FAD [24].

### 2.6. Steady-State Kinetics

In order to elucidate the kinetic mechanism of TGR, initial velocity data were obtained by varying the concentration of both NADPH and GSSG [25]. For the determination of the kinetic mechanism with GSSG as the substrate, concentrations up to 60 *μ*M of the disulfide were used in order to avoid the strong substrate inhibition observed at moderate or high concentrations of GSSG. This decision is warranted because the initial portion of the saturation curve is very sensitive to discriminate between sequential and ping-pong kinetic mechanisms [25]. A global fitting of data through nonlinear regression to the rate equation for either a ping-pong bi bi (see (1)) or ordered bi bi (see (2)) kinetic mechanisms in the absence of products was then performed.

(1) v=VmABKmBA+KmAB+AB

(2) v=VmABKiaKmB+KmBA+KmAB+AB

where A represents NADPH, B corresponds to either GSSG or DTNB, and *Km*_A_ and *Km*_B_ are the corresponding Michaelis-Menten constants, while* Kia* represents the dissociation constant for A. The inhibitory ability of NADP^+^ on the GSSG reductase activity of TGR was determined by analyzing its effect on the initial velocities under steady-state conditions. NADP^+^ was incubated in buffer A with NADPH and GSSG under nonhysteretic conditions and the reaction was started by adding a small enzyme aliquot. Data obtained in the presence of the product NADP^+^ as the inhibitor were fitted to either a competitive (see (3)) or an uncompetitive (see (4)) model of inhibition:

(3) v=VmAKmA1+P/Kis+A1+KmB/B

(4) v=VmBKmB+B1+KmA/A1+P/Kii

where A and B are defined as above and P stands for NADP^+^, while *K*_*is*_ and *K*_*ii*_ represent slope and intercept inhibition constants, respectively. These two latter equations were derived by using the King-Altman method [26] for a ping-pong bi bi kinetic mechanism in which NADP^+^ acts as a dead-end inhibitor through the formation of a complex with the unmodified form of the enzyme. Finally, to gain insight into the GSSG-dependent substrate inhibition of TGR, initial velocity data obtained over a broad range of both NADPH and GSSG concentrations were fitted to

(5) $$\begin{array}{c} v=\frac{{Vm}_{1}\left[A\right]\left[B\right]+{Vm}_{2}\left[A\right]{\left[B\right]}^{2}/{Km}_{B^{\prime }}}{{Km}_{B}\left[A\right]+{Km}_{A}\left[B\right]+\left[A\right]\left[B\right]+\left[A\right]{\left[B\right]}^{2}/{Km}_{B^{\prime }}+\left[A\right]{\left[B\right]}^{3}/{Km}_{B^{\prime }}K_{i}+{Km}_{A}{\left[B\right]}^{2}/{Km}_{B^{\prime }}} \end{array}$$

where A, B, *Km*_A_, and *Km*_B_ are defined as above and* Vm*_1_ represents the catalytic pathway followed by the enzyme at low concentrations of GSSG, while* Vm*_2_ corresponds to the alternative minor catalytic pathway obtained at high concentrations of GSSG. *Km*_B_' is a second* Km* value for substrate B, while* Ki* is the inhibitor constant for GSSG acting as inhibitor (see the corresponding model under discussion). An initial estimate for the* K*i value was obtained by plotting initial velocity data obtained over a broad range of GSSG concentrations in a semilog fashion [27] and the following equation:

(6) $$\begin{array}{c} K_{i}=S_{1}+S_{2}-4S_{m} \end{array}$$

where S_m_ represent the concentration of substrate at the maximum point of the curve, while S_1_ and S_2_ correspond to the substrate concentration at the two points where velocity is half that at the maximum.

In those kinetic experiments carried out at high concentrations of GSSG, the magnitude of the apparent lag time observed in the full time progress curves was estimated as described elsewhere [28].

### 2.7. Statistical Analysis

Fitting of data to the different velocity equations was made by nonlinear regression analysis using Sigma Plot software. No weighting of data was applied. Kinetic parameters are given as mean ± standard deviation.

### 2.8. Model Discrimination Analysis

When the initial velocity patterns did not allow a clear distinction between alternative kinetic mechanisms, a model discrimination analysis was needed. The following rules were used in the data analysis.

(I) The initial selection of a particular kinetic model was based on visual inspection of the corresponding double-reciprocal plot. When this preliminary analysis did not lead to a clear model discrimination, data were fitted to both alternative velocity equations.

(II) If the two alternative kinetic models had equal or very similar *χ*^2^ values, then the model consistent with additional kinetic evidence (i.e., inhibitory patterns) was chosen as the most plausible.

### 2.9. Global Data Fitting Procedure

In order to compare the experimental full progress curves obtained under different concentrations of NADPH, GSSG, and enzyme, with that predicted by the mechanistic model (see discussion), a curve fitting procedure was performed. In this case the fitting procedures were carried out with the Dynafit software [29] version 4. The following conditions were fixed.

(i) In all cases, the best set of rate constants pertaining to the ping-pong reaction cycle were fixed (see Supplementary Materials), representing ten microscopic rate constants (reactions 1 to 6 of the model).

(ii) In the fitting procedure of the full progress curves obtained at moderate or high concentrations of GSSG, two rate constants were allowed to be fitted in order to find the best fit values. One of such constants pertains to a reaction associated with the reversible binding of GSSG to the inhibitory site (reactions 7 and 9 of the model), while the other microscopic rate constant was chosen from any of the two reactions responsible for the atypical full progress curves (reactions 10 and 12 or reactions 11 and 13, see Figure 11). The same conditions were used in those cases where a global fitting of several full progress curves was carried out.

(iii) For long full time courses, the best fitting value for either GSSG or enzyme concentrations, or both, was also searched, allowing a variation of ±10% of the experimental value.

### 2.10. In Silico Analysis

Simulation of the full time courses of TGR was performed with the Dynafit software [29] version 4. The particular conditions used in the modelling are described in Supplementary Materials.

### 2.11. Docking Analysis

For molecular docking, the protein structure of* S. mansoni* TGR (PDB code 2X8C) was obtained from the RCSB Protein Data Bank (http://www.rcsb.org). The structure of GSSG was recovered from the* Homo sapiens* glutathione S-transferase *μ*2 (PDB code 1YKC) using the Hic-up server (http://xray.bmc.uu.se/hicup/). The structures of TGR and GSSG were processed with AutoDockTools (ADT) version 1.5.4 (http://mgltools.scripps.edu) [30]. Essential hydrogen atoms and Kollman united atom charges were added to the protein and then saved in PDBQT file format, for input into Auto Dock Vina version 1.1.2 (http://vina.scripps.edu) [31]. For TGR, the search space was defined as a grid box of size 24 × 20 x 20 Å with a grid spacing of 0.375 Å, centered on the putative binding site.

## 3. Results

### 3.1. Steady-State Kinetic Study of TGR

**Figure 1:**
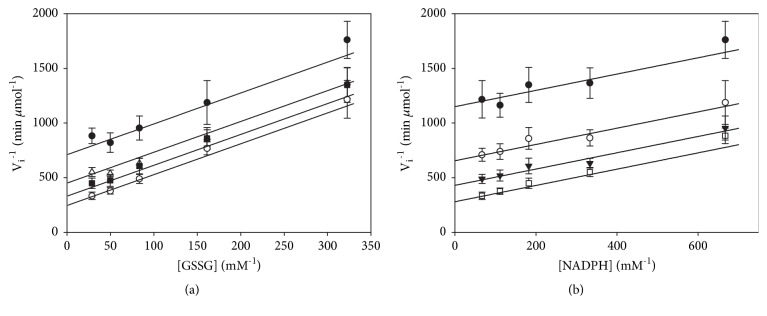
**Initial velocity patterns of* T. crassiceps* TGR**. Enzyme assays were carried out as described under Materials and Methods at 25°C and pH 7.8 in the presence of low concentrations of GSSG. (a) GSSG as the variable substrate at the following fixed concentrations of NADPH: (●) 1.5 *μ*M; (Δ) 3*μ*M; (■) 5.5 *μ*M; (○) 15 *μ*M. (b) NADPH as the variable substrate at the following fixed concentrations of GSSG: (●) 3.1 *μ*M. (○) 6.2 *μ*M; (▼) 12 *μ*M; (□) 36 *μ*M. The final enzyme subunit concentration was 6.1 nM. Continuous lines were obtained from the corresponding double-reciprocal form of equation (1) using the parameters resultant from the global fitting of data (each point represents mean ± standard deviation (n=6).

**Figure 2:**
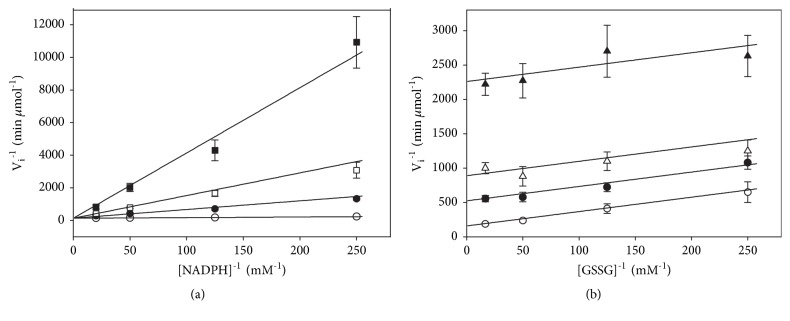
**Product inhibition patterns of* T. crassiceps* TGR by NADP** ^**+**^. Enzyme assays and incubation conditions were as described under Materials and Methods. (a) NADPH as the variable substrate at a constant GSSG concentration (70 *μ*M) and the following fixed concentrations of NADP^+^: (○) 0; (●) 0.5 mM; (□) 1.5 mM; (■) 5 mM. Continuous lines were obtained from the corresponding double-reciprocal form of equation (3) using the parameters resulting from the global fitting of data. (b) GSSG as the variable substrate at a constant NADPH concentration (20 *μ*M) and the following fixed concentrations of NADP^+^: (○) 0; (●) 0.8 mM; (Δ) 1.6 mM; (▲) 5 mM. Continuous lines were obtained from the corresponding double-reciprocal form of equation (4) using the parameters resulting from the global fitting of data. In all of these inhibition experiments, the final concentration of enzyme subunit was 6.1 nM. Each point represents mean ± standard deviation (n=6).

**Figure 3:**
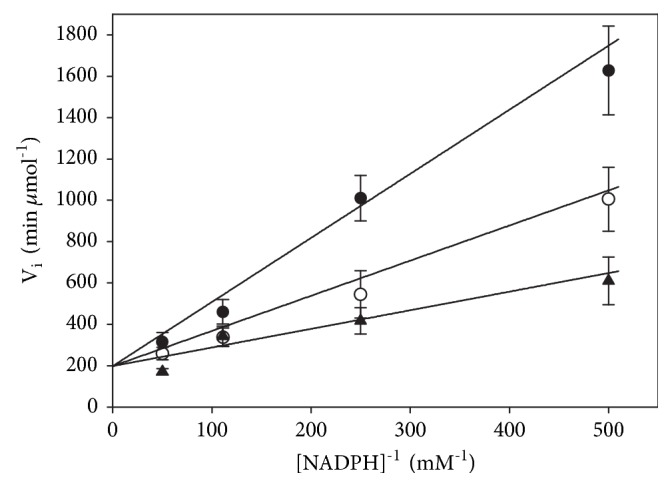
**Double-reciprocal plot of the initial velocity data**. Each line was obtained by varying the concentration of both NADPH and GSSG such that [GSSG] = *χ* [NADPH]. The corresponding *χ* factor values were as follows: (●) 1; (○) 2.5; (▲) 8. Final concentration of enzyme subunit was 5.3 nM. Lines represent the result of the global adjustment of initial velocity data to the corresponding equation [27]. Each point represents mean ± standard deviation (n=6).

Figure 1 shows the results of the initial velocity experiments presented as double-reciprocal plots. With either GSSG (Figure 1(a)) or NADPH (Figure 1(b)) as the variable substrate a parallel pattern of lines was obtained, suggesting TGR follows a ping-pong bi bi kinetic mechanism. When GSSG was replaced with DTNB, an artificial substrate indicative of TR activity, identical parallel double-reciprocal plots were obtained (data not shown). The global fitting procedure of the initial velocity data to either a ping-pong or an ordered sequential kinetic mechanism resulted in essentially identical kinetic parameters. However, fitting of data to the rate equation for an ordered mechanism required a too low* K*ia value (4.98 × 10^−17^*μ*M) with a very high variation coefficient (7.4 × 10^17^%). To confirm the ping-pong kinetic model suggested by the initial velocity patterns, a product inhibition study with NADP^+^ was performed. The results are shown in Figure 2. With NADPH as the variable substrate, double-reciprocal plots showed an intersecting pattern (Figure 2(a)); however, the graphical analysis of the data did not allow a clear distinction between the competitive or the mixed-type modes of inhibition. Similarly, the statistical results of the global fitting procedure showed very similar *χ*^2^ values (1.49 versus 1.4 for the competitive or the mixed-type inhibition, respectively). In order to elucidate this point, the effect of NADP^+^ on the initial velocity patterns, with GSSG as the variable substrate, was also analyzed. The resultant double-reciprocal plots showed a parallel lines pattern, strongly suggesting an uncompetitive inhibition (Figure 2(b)). Clearly, the above inhibition patterns are not the expected ones for a typical ping-pong bi bi kinetic mechanism, in which NADP^+^ is expected to act as a mixed-type inhibitor against NADPH and as a competitive inhibitor against GSSG, respectively [25, 32]. To clarify these apparently contradictory results an additional experiment, in which the concentrations of both NADPH and GSSG were varied together while maintaining their concentrations at a constant ratio was performed. The results of such experiment revealed linear double-reciprocal plots (Figure 3), consistent with a ping-pong bi bi kinetic mechanism [25]. Hence, the unexpected inhibition patterns obtained with NADP^+^ are due to the formation of a dead-end complex between NADP^+^ and the form of the enzyme to which NADPH binds. As a corollary of such proposal, it is expected that the dissociation of NADP^+^ from the reduced form of the enzyme during the normal catalytic cycle would be an irreversible process. To test this prediction, the effect of NADP^+^ at a low concentration on the initial velocity pattern, with GSSG as the variable substrate, was analyzed. The results revealed no effect of NADP^+^ on the slope of the double-reciprocal plots (data not shown), confirming the lack of a reversible connection during the NADP^+^ dissociation step. Hence, it can be concluded that the inhibition of the product NADP^+^ on the GSSG reductase activity of TGR is the result of the formation of a dead-end complex with the unmodified enzyme, explaining the competitive inhibition pattern of NADP^+^ with NADPH as the variable substrate. In Table 1 the kinetic parameters *K*_m_, *k*_cat_ as well as the specificity constant *k*_cat_/*K*_m_ for both NADPH and GSSG of* T. crassiceps* TGR are summarized. These values are consistent with those previously reported [14].

Reduced glutathione was a very poor inhibitor product. At 8 mM GSH, barely 10% inhibition was detected. Between 1 and 4 mM GSH, a moderate but consistent activating effect (up to 25%) was observed. Due to the micromolar concentrations of GSSG present in the GSH stock solutions, its effect as an inhibitor product was not further explored.

### 3.2. GSSG-Dependent Substrate Inhibition of TGR

**Figure 4:**
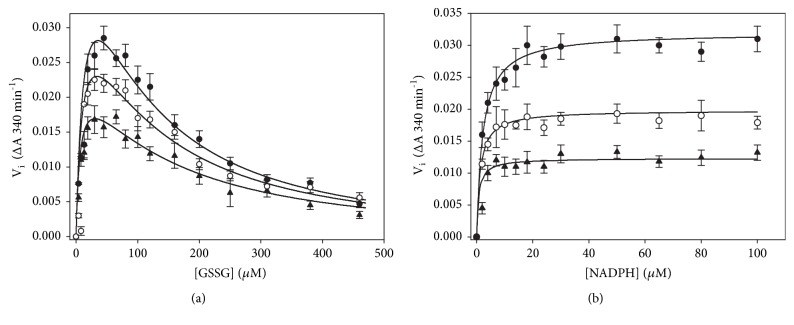
**Dependence of initial velocities of* T. crassiceps* TGR on either GSSG (a) or NADPH (b) concentrations. **Data were obtained as described under Materials and Methods. (a) GSSG as the variable substrate at the following constant concentrations of NADPH: (▲) 3 *μ*M; (○) 8 *μ*M; (●) 40 *μ*M. (b) NADPH as the variable substrate at the following constant concentrations of GSSG: (▲) 4 *μ*M; (○) 10 *μ*M; (●) 35 *μ*M. In all the enzyme assays, the final concentration of enzyme subunit was 14 nM. In order to avoid overlapping of data, only results obtained at three constant concentrations of the corresponding fixed substrate is shown. Continuous lines were obtained from equation (5) using the parameters resulting from the global adjustment of data. Each point represents mean ± standard deviation (n=6).

In the presence of moderate or high concentrations of the substrate GSSG, the disulfide reductase activity of* T. crassiceps* TGR is strongly inhibited. Such inhibition is concomitant with the appearance of the hysteretic-like progress curves [14], suggesting both phenomena could be mechanistically related.  Figure 4 shows the saturation curves obtained with either GSSG or NADPH as the variable substrate in a broad range of concentrations. A strong inhibitory effect with GSSG at any constant concentration of NADPH was observed (Figure 4(a)); in contrast, no inhibition with NADPH was detected, even at concentrations as high as 30 times *K*_m_ (Figure 4(b)). To warrant the kinetic analysis of the initial velocity data obtained at high concentrations of GSSG in the presence of the atypical time courses, it was necessary to perform* in silico* simulations using the mechanism-based model (see discussion) either in the presence or in the absence of the reactions involved in the generation of the hysteretic-like full time courses. The results of the simulations revealed no significant difference when the initial velocity data obtained under the two above-mentioned conditions were compared (Fig.  S1). Therefore, it can be concluded that, in the initial stages of the reaction carried out at a high GSSG concentration, steady-state conditions can be assumed. Thus, the initial velocity data shown in Figure 4 were fitted to (5) to obtain the kinetic parameters* V*m_2_, *Km*′_B_, and* K*i. Results of the global fitting procedure revealed the* V*m_2_ value is barely 2.5% of* V*m_1_. As regards* K*i, the resultant figure of 331 ± 104 *μ*M is consistent with the value of 310 ± 47 *μ*M obtained by (6). A summary of these kinetics parameters is shown in Table 1.

### 3.3. Effect of NADPH on the Hysteretic-Like Kinetic Behavior of TGR

**Figure 5:**
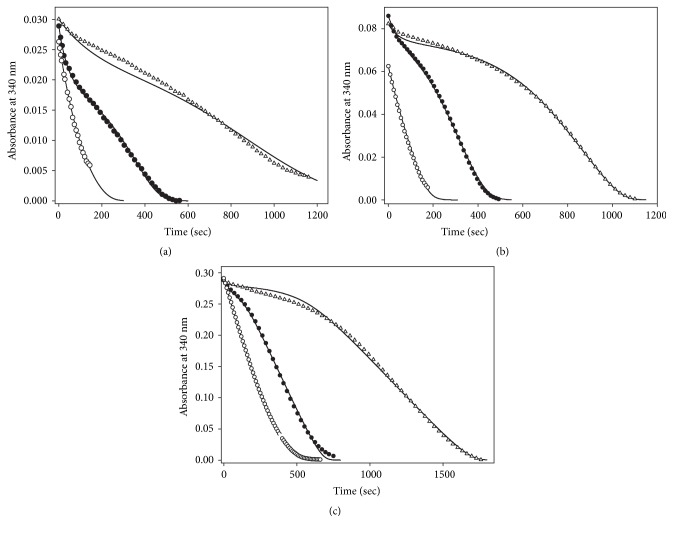
**Full time progress curves of* T. crassiceps* TGR as a function of both NADPH and GSSG concentration**. Enzyme assays and incubation conditions were as described under Materials and Methods. The particular concentration of NADPH, GSSG, and enzyme used in the corresponding enzyme assays were as follows: Panel (a): (○) 4.2 *μ*M NADPH, 5 *μ*M GSSG, 14.7 nM TGR; (●) 4.7 *μ*M, 270 *μ*M GSSG, 11.5 nM TGR; (Δ) 4.9 *μ*M NADPH, 500 *μ*M GSSG, 11.5 nM TGR. Panel (b): (○) 10.1 *μ*M NADPH, 20 *μ*M GSSG, 14.7 nM TGR; (●) 13.9 *μ*M NADPH, 200 *μ*M GSSG, 11.5 nM TGR; (Δ) 13.4 *μ*M NADPH, 510 *μ*M GSSG, 11.5 nM TGR. Panel (c): (○) 47 *μ*M NADPH, 120 *μ*M GSSG, 11.5 nM TGR; (●) 46.5 *μ*M NADPH, 200 *μ*M GSSG, 11 nM TGR; (Δ) 46 *μ*M NADPH, 310 *μ*M GSSG, 6.6 nM TGR. Continuous lines represent fitting of experimental data points to the mechanism-based model using the best set of rate constants (see Materials and Methods for details of the conditions used in the fitting procedure).

**Figure 6:**
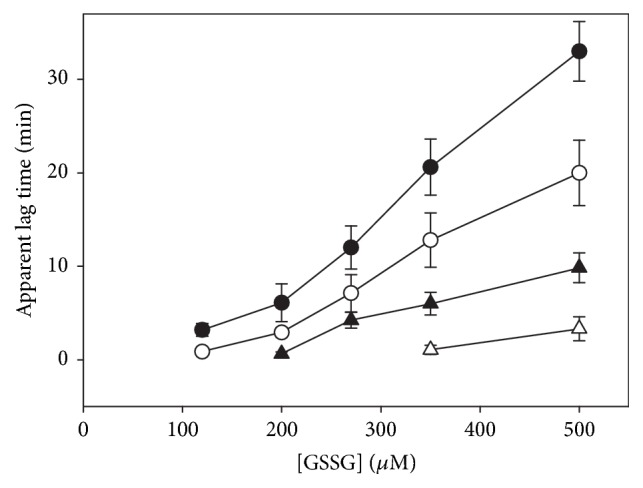
**Dependence of the apparent lag time on the concentration of both NADPH and GSSG**. The magnitude of the relaxation time was estimated as described under Materials and Methods from full time courses performed at 25°C and pH 7.8 at the following NADPH concentrations: (Δ) 5.5 *μ*M; (▲) 9 *μ*M; (○) 15.5 *μ*M; (●) 50 *μ*M. Each point represents mean ± standard deviation (n=6).

To analyze the effect of NADPH on the atypical kinetic behavior of* T. crassiceps* TGR, enzyme assays of GSSG reductase activity under a variety of initial concentrations of NADPH, GSSG, and enzyme were carried out.  Figure 5 shows representative full progress curves. At low concentrations of both substrates (Figures 5(a) and 5(b), left traces), conventional profiles of NADPH consumption were observed. By increasing the concentration of either NADPH or GSSG, however, complex profiles of the progress curves resulted (Figures 5(a) and 5(b), middle traces), which were particularly noticeable between 100 *μ*M and 300 *μ*M GSSG. In such profiles three kinetic stages of NADPH consumption can be discerned, a first burst-like stage in which the rate of NADPH consumption was relatively fast, followed by a second stage where a temporary inhibition of the GSSG reductase activity was evident, and a final third stage in which the enzyme activity was regained, leading the reaction into an apparent steady-state condition. The relative amplitude, as well as the duration of the first and second stages of the reaction, was strongly dependent on the concentration of both substrates as well as the enzyme concentration. At 4.7 *μ*M NADPH and 270 *μ*M GSSG (Figure 5(a), middle trace) the temporary inhibition stage was already detectable, and the initial fast stage of NADPH oxidation was clearly observable. By contrast, at 46.5 *μ*M NADPH and 310 *μ*M GSSG (Figure 5(c), right trace), the initial fast consumption of NADPH was barely detectable and the temporary inhibition stage was extended over ten minutes. At any NADPH concentration where the GSSG-dependent substrate inhibition is observed, increasing the initial concentration of GSSG in the reaction mixture resulted in a decrease in both the initial velocity and the relative amplitude of the first stage, concomitant with a significant rise in the duration of the inhibited stage. An identical effect was obtained by increasing the concentration of NADPH at a constant but high enough GSSG concentration.  Figure 6 shows the dependence of the apparent lag time on the concentration of GSSG at different NADPH concentrations. Clearly, its magnitude is determined by the concentration of both substrates. The complex full progress curves were observed at any NADPH concentration when the GSSG concentration was high enough.

By increasing the enzyme concentration in the reaction mixture under conditions in which an apparent lag phase was observed resulted in a significant rise in the amplitude of the initial fast stage, concomitant with a decrease in the lag time as previously reported [14]. Attempts to fit the full time course data to the single exponential equation derived for simple hysteretic kinetics [28] were unsuccessful.

**Figure 7:**
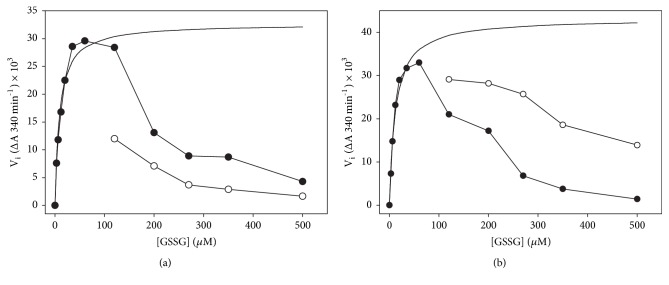
**Dependence of the initial and the apparent steady-state velocities on both NADPH and GSSG concentration**. Initial (●) and apparent steady-state (○) velocities were obtained from full progress curves by varying the concentration of GSSG at the following initial concentrations of NADPH: (a) 9 *μ*M; (b) 50 *μ*M. Continuous spline lines represent the initial velocities predicted by the rate equation for a ping-pong bi bi kinetic mechanism in the absence of substrate inhibition. Each point represent the average of six experiments.

The above results strongly suggest that, under conditions in which complex time courses were observed, continuous changes in the relative abundance of both active and inactive complexes of the enzyme were occurring. It is worth noting that, under any condition in which an atypical full progress curve was observed, the magnitude of the velocity calculated from the maximal slope at the apparent steady-state segment of the curve was significantly lower than that predicted from the rate equation for a ping-pong bi bi kinetic mechanism (Figure 7), both at a low (Figure 7(a)) and at a high NADPH (Figure 7(b)) concentration. Even when the accumulation of NADP^+^ and the depletion of the substrates were taken into account, the same results were obtained. Thus, in the apparent steady-state segment of the progress curves, the inhibition of TGR by GSSG was still present, and the degree of inhibition depended on the initial concentrations of both NADPH and GSSG.

### 3.4. Effect of GSSG on Trx Reduction

**Figure 8:**
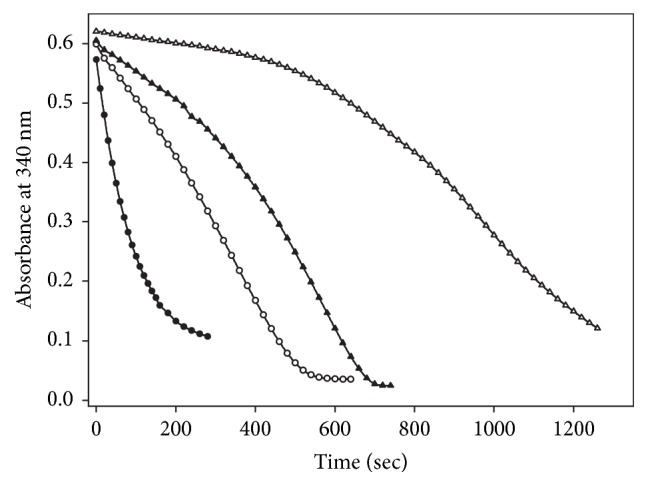
**Effect of GSSG on the reduction of human Trx by* T. crassiceps* TGR**. Reaction mixtures were prepared in buffer A by mixing Trx with 100 *μ*M NADPH either in the presence or in the absence of 2 mM GSSG. After two minutes, enzyme was added to start the reaction. The final volume of the reaction mixture was 120 *μ*L. (Δ) 2 mM GSSG, no trx; (●) 150 *μ*M Trx, no GSSG; (▲) 2 mM GSSG plus 60 *μ*M Trx; (○) 2 mM GSSG plus 150 *μ*M Trx. The final concentration of enzyme was 37 nM.

When the alternative biological disulfide Trx was assayed at relatively high concentrations (up to 170 *μ*M), no evidence for substrate inhibition or hysteretic kinetics was observed [14]. Hence, it can be concluded that both the substrate inhibition and the apparent hysteretic kinetic of TGR are due only to GSSG. However, the addition of a high concentration of GSSG to the assay mixture for TR activity resulted in inhibition (Figure 8). With Trx as the alternative substrate, the profile of the full time course obtained in the presence of 2 mM GSSG was similar to those described for the reduction of GSSG under hysteretic conditions, showing a clear temporary inhibition stage (Figure 8). By increasing the concentration of Trx in the reaction mixture, however, the magnitude of the lag time was significantly shortened.

### 3.5. Docking Analysis with GSSG

**Figure 9:**
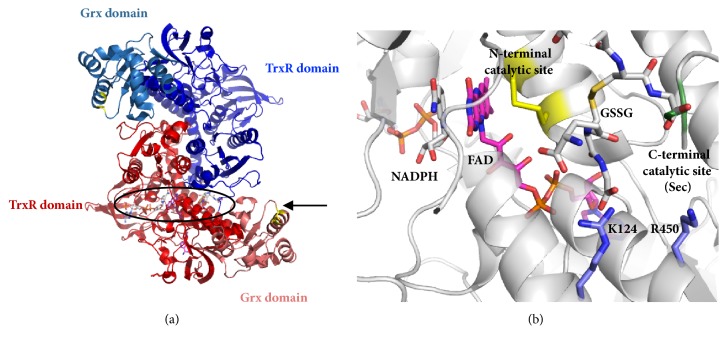
(a) Dimeric structure of* S. mansoni* TGR as derived from X-ray crystallography. Monomers of the enzyme are shown in either blue or red and the location of the glutaredoxin domains is indicated. The enzyme was placed such that the region in which both the NADPH and the FAD binding sites, enclosed in an oval, could be viewed. (b) Amplified view showing details of the potential binding site for GSSG as inhibitor as derived from docking studies. The reducing substrate NADPH, as well as the prosthetic group FAD and the redox active disulfide (in yellow), are shown in stick. The catalytically essential selenocysteine residue of the partner subunit is shown in green.

**Figure 10:**
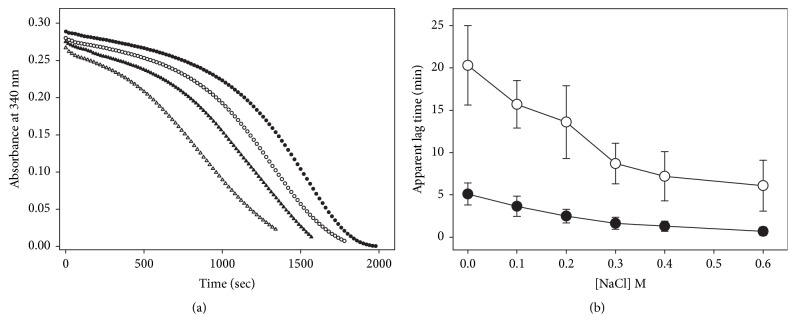
**Effect of ionic strength on the apparent lag time of* T. crassiceps* T**GR. Enzyme assays and incubation conditions were as described under Materials and Methods. (a) Representative full progress curves of GSSG reductase activity determined in the presence of 50 *μ*M NADPH and 600 *μ*M GSSG at the following concentrations of NaCl: (●) control without NaCl; (○) 0.2 M NaCl; (▲) 0.4 M NaCl; (Δ) 0.6 M NaCl. To avoid overlapping of traces, curves were displaced vertically. (b) Dependence of the apparent lag time on NaCl concentration. Enzyme assays were carried out in the presence of either 300 *μ*M GSSG (●) or 600 *μ*M GSSG (○). Each point represents mean ± standard deviation (n=6).

From the X-ray crystallographic analysis of* S. mansoni* TGR a potential zone for GSSG binding was revealed [8]. It is located at the* si* face of FAD and is characterized by a high density of positively charged residues (K124, K128, R450, and R454), giving a surface electrostatic potential which is more similar to the corresponding site on GR than that of the homologous TR [8]. Docking analysis with TGR from both* S. mansoni* [10] and* F. gigantica* [16] revealed the feasibility for GSSG binding at this putative alternative site. The results of such studies suggest that GSSG is able to interact with the positively charged residues K124 and R450 through electrostatic interactions. We have confirmed these results with TGR from* T. crassiceps* and the associated binding enthalpy was estimated as – 7.1 kcal mol^−1^. This figure is significantly lower than the value of – 24.6 kcal mol^−1^ obtained by isothermic titration calorimetry for the binding of GSSG to the active site of human GR [33], suggesting a weaker interaction of GSSG at the corresponding site on TGR. In this sense, the value of 385 *μ*M for* K*i obtained in the present work from the substrate inhibition data is consistent with such result.  Figure 9(b) shows the location on the enzyme at which GSSG is potentially bound as inhibitor. Its disulfide bond is located midway between the redox active dithiol of the enzyme and the catalytically essential selenocysteine residue of the neighbor subunit. Assuming that electrostatic interactions are involved in the GSSG binding at the putative inhibitory site, then it can be guessed that the ionic strength of the medium could modify the atypical full progress curves of TGR. Experiments to test such prediction were carried out.  Figure 10 shows the effect of NaCl on both the atypical profile of the hysteretic-like progress curves and on the apparent lag time. Clearly, by increasing the ionic strength in the reaction mixture a significant decrease in the magnitude of the apparent lag time, concomitant with a slight increase in the amplitude of the burst-like stage, was obtained. Thus, the involvement of electrostatic interactions in GSSG binding at the inhibitory site is strongly suggested. Although the diminution in the size of the apparent lag time due to NaCl resulted in an increase in the amplitude of the burst-like stage, the initial velocities measured were still far below that expected in the absence of inhibition. This is the result of an inhibitory effect by NaCl on the reductase activity overlapped with its ability to modify the lag time. Such inhibition was confirmed by analyzing the effect of NaCl on the GSSG reductase activity of TGR at a low concentration of GSSG (80 *μ*M). In the range from 50 mM to 600 mM NaCl, an inhibition up to 70% of the initial velocity was observed.

## 4. Discussion

TGR represents an atypical case in the disulfide reductase family of enzymes. Although the enzyme retains the homodimeric nature which is common to this set of oxidoreductases, it is outstanding in tertiary structure, total number of redox centers, and wide substrate specificity. Thus, in TGR a Grx-like domain has been appended to the N-terminal end of the animal TR module, conferring to the enzyme additional catalytic abilities, notably the reduction of GSSG at significant rates, as well as catalysis of thiol-disulfide exchange reactions, including deglutathionylation of mixed disulfides protein-glutathione [1, 4, 6]. Furthermore, the dithiol/disulfide motif of the Grx-like domain of TGR adds to the FAD prosthetic group and the N- and C-terminal redox centers of the TR module, giving to the enzyme the potential ability to store up to eight reducing equivalents in its maximal reduction state. In addition to the above features, TGR displays unusual full time courses of enzyme activity at moderate or high concentrations of GSSG [5, 14–17], which have been considered as hysteretic behavior [5, 14]. To explain such atypical kinetics of TGR, a model based on covalent modification through glutathionylation of the structural cysteine residues 88 and 354 of the enzyme from* E. granulosus* [5] was proposed. However, such model is not supported by the experimental observations. In this sense, TGR from the flukes* F. hepatica* [15] and* S. mansoni* [7] also shows atypical full time progress curves of reductase activity at high concentrations of GSSG, even though they lack the two above noted cysteine residues. An alternative hypothesis, based on the dithiol/disulfide redox motif of the Grx-like domain, was put forward to explain the hysteretic behavior of the enzyme [6]. Thus, when the C-terminal cysteine residue of the dithiol/disulfide redox center of the Grx domain (Cys34 of* E. granulosus* TGR) was replaced with serine, the lag time observed at a high concentration of GSSG was fully abolished. However, by decreasing the protein concentration of the mutant enzyme in the reaction mixture, the hysteretic-like progress curve was regained [6]. In these two hypotheses, the role of NADPH in the hysteretic behavior of TGR was not considered.

The results described in the present work show that TGR from* T. crassiceps* cysticerci follows a ping-pong bi bi kinetic mechanism with the NADPH/GSSG couple as the substrates, in which dissociation of NADP^+^ from the enzyme during the catalytic cycle is an irreversible event. Interestingly, the inhibition patterns obtained with NADP^+^ are consistent with a variant of the ping-pong bi bi kinetic mechanism typical for two-site enzymes [34–36]. According to such mechanism, binding of the substrates on the enzyme occurs at separate sites under rapid equilibrium conditions, requiring a mobile carrier in order to transfer the corresponding chemical group between both substrate binding sites. The three-dimensional structure of* S. mansoni* TGR [8, 9] is consistent with such proposal. In this enzyme, the role of the mobile carrier is played by the C-terminal end of the neighbor subunit, where the essential selenocysteine residue is located. Further, in a two-site enzyme, dissociation of the first product (i.e., NADP^+^) can occur either before or after binding of the second substrate (i.e., GSSG), explaining the atypical inhibition patterns observed with* T. crassiceps* TGR. Thus, TGR can be considered as an additional example of enzymes where the active site is split into halves.

On the other hand, the initial velocity data obtained in a broad range of GSSG concentrations revealed a strong substrate inhibition (Figure 4(a)). The overlap of the latter with the atypical progress curves could, in principle, reject a kinetic analysis of the initial rate data by the steady-state formalism. However, the results of the* in silico* simulations performed either in the presence or in the absence of the reactions responsible for the unusual kinetic behavior of TGR (see below) revealed such analysis is warranted. As shown in Fig.  S1, differences in the initial velocity data are observed at GSSG concentrations above 50 *μ*M, reaching a maximum (about 8%) at the highest concentrations of GSSG, both at a low and at a high NADPH concentration. Such differences are in the range of the experimental uncertainty obtained in the determination of initial velocities.

As regards the hysteretic-like full progress curves of TGR observed at moderate or high concentrations of GSSG, the results obtained in the present work revealed the presence of an additional complexity. Thus, the enzyme assays performed at low concentrations of the electron donor NADPH showed an initial burst-like stage, followed by the lag stage. The initial stage of fast NADPH consumption is barely noticeable in the enzyme assays carried out in the presence of high concentrations of both GSSG and NADPH [5, 6, 14, 16, 17]. Due to its minor contribution to the full progress curves of reductase activity under these conditions, such initial stage was not considered in any previous work dealing with the atypical kinetic behavior of TGR. As was demonstrated in the present work, the relative contribution of both the burst-like and the lag-like stages to the time course depends on the concentration of both NADPH and GSSG.

In order to build a comprehensive model to explain the atypical kinetic behavior of TGR, the following observations regarding the normal performance of the enzyme must be stated. These are based on all the kinetic and structural evidence available.  Figure 9 shows a full view of dimeric TGR (Figure 9(a)) as well as a detailed view (Figure 9(b)) at the TR module region where both the NADPH binding site and the N-terminal dithiol/disulfide redox active motif are located.

(i) In the GSSG reductase activity of TGR, the Grx-like domain of the enzyme plays an essential role [1, 4, 7]. Hence, its dithiol/disulfide redox motif must be in the reduced state. Thus, in addition to the reduced redox centers typical of the high molecular weight TR, in TGR additional reducing equivalents are needed in order to catalyze GSSG reduction. Therefore, during the catalytic cycle the enzyme must oscillate between states with a reduction degree higher than the two-electron reduced state. Recent work with* S. mansoni* TGR [9] supports the existence of the four-electron (EH_4_) reduced species as an intermediary in its normal functioning.

(ii) Natural variants of the enzyme (e.g., mouse testes TGR) in which the C-terminal cysteine residue of the redox motif at the Grx-like domain is absent are fully functional in GSSG reduction [1]. This fact strongly suggests that in the GSSG reduction pathway by TGR, the Grx-like domain is able to catalyze only a thiol-disulfide exchange reaction with either GSSG or protein-glutathione mixed disulfides. In this proposal, the N-terminal cysteine residue of such motif will be involved in the nucleophilic attack on the disulfide bond of GSSG, leading to the formation of a glutathione-enzyme mixed disulfide [4]. Experiments carried out with TGR mutants of both* E. granulosus* and* S. mansoni*, in which either the N-terminal or the C-terminal cysteine residue of the Grx redox motif was replaced [6, 7], support such proposal. In this sense, it is worth noting that, for typical Grx, only the N-terminal redox active cysteine is required in thiol-disulfide exchange reactions [37, 38].

(iii) As with the related TR, both subunits of TGR are required during the catalytic cycle of the enzyme. Thus, in order to regenerate the reduced state of the nucleophilic cysteine of subunit A, electrons must be shuttled from the selenol-thiol redox center, located at the C-terminal end of subunit B [9]. However, as was revealed by the X-ray crystallographic studies with* S. mansoni* TGR (Figure 9(a)), the two redox centers are distant from one another [8, 9]. Thereby, in order for the electron transfer to take place, a large conformational change of the C-terminal arm of the enzyme is needed. Molecular dynamic simulations suggest such change is feasible [10]. In a recent work with* E. granulosus* TGR it is proposed that the Grx-like domain of the enzyme participates in the conformational change during the aforementioned electron transfer [37].

(iv) The X-ray crystallographic structures of both* S. mansoni* [8] and* E. granulosus* [37] TGR have revealed the existence of a potential second site for GSSG binding, located on the TR module of the enzyme at the FAD binding domain (Figure 9(b)). This region is characterized by a high charge density whose electrostatic potential is similar to the binding site for GSSG on GR [8]. Hence, the putative interaction of GSSG on this site must involve electrostatic interactions. The results dealing with the effect of the ionic strength on the apparent lag time reported in the present work support the latter conclusion, while the docking studies have revealed that such interaction is both structurally and thermodynamically feasible [10, 16].

Regarding the complex kinetic behavior of TGR, the following experimental facts must be considered.

(i) The profiles of the full progress curve of NADPH consumption and the magnitude of the apparent lag time are dependent on the concentration of the substrates NADPH and GSSG. Thus, the amplitude of both the initial burst-like stage and the inhibited segment of the progress curves can be modified by changing the initial concentration of the substrates in the reaction mixture.

(ii) Both the magnitude of the apparent lag time and the profile of the full progress curves of TGR are not those expected for a typical hysteretic enzyme. For the latter, the reported lag times are in the range of seconds or a few minutes and the shape of the curve in the transition zone is due to a single exponential transition [28, 38–41].

(iii) The presence of disulfide reducing reagents (e.g., GSH, DTT, cysteine) at micromolar concentrations in the reaction mixture lead either to a decrease in the magnitude of the apparent lag time or its full abolition [5, 14, 15]. In this sense, in the presence of 50 *μ*M GSH saturation curves with GSSG as the variable substrate was fully hyperbolic. Up to a concentration of 300 *μ*M of the disulfide no inhibition was observed (data not shown). Such evidence strongly suggests that in the atypical kinetic behavior of TGR thiol/disulfide exchange reactions are involved.

(iv) The concentration of the enzyme in the reaction mixture is also critical for the presence or absence of the atypical progress curves as well as for the amplitude of the initial fast stage of the reaction. The higher the enzyme concentration, the lesser the magnitude of the apparent lag time [5, 14].

**Figure 11:**
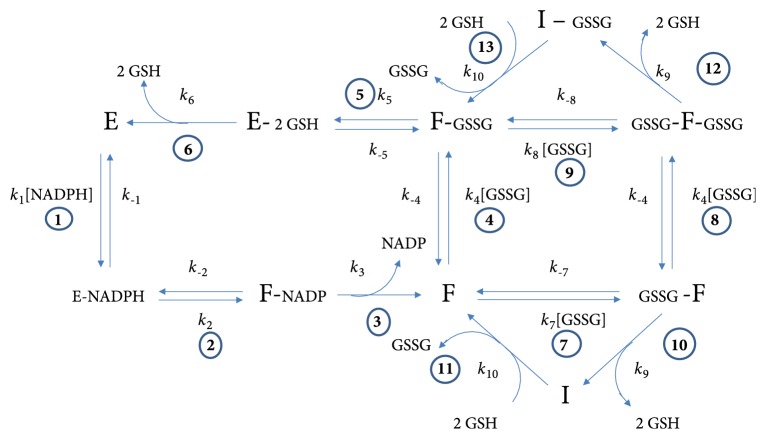
**Mechanism-based model for GSSG reductase activity of* T. crassiceps* TGR**. Reactions 1 to 6 pertain to the ping-pong bi bi kinetic mechanism; reactions 7 to 9 correspond to the reversible inhibitory branch in which the substrate GSSG acts as noncompetitive inhibitor. Reactions 10 and 12 represent residual GSSG reductase activity of the inhibited complexes while reactions 11 and 13 correspond to reactivation of the inactive intermediaries I and I-GSSG by GSH. Reactions 1, 4, and 8 of the model correspond to those segments working under rapid equilibrium conditions.

Based on all the above kinetic and the structural evidence on TGR, a mechanism-based model was built to explain its atypical kinetic properties (Figure 11). The major features of the model are briefly described:

(i) According to the results obtained in the present work, the normal catalytic cycle of the enzyme (reactions 1 to 6 in Figure 11) is based on a two-site ping-pong bi bi kinetic mechanism, in which both NADPH and GSSG bind at different sites under rapid equilibrium conditions. Such sites are located at the TR module and the grx-like domain of the enzyme for NADPH and GSSG, respectively, as derived from the X-ray crystallographic studies of* S. mansoni* [8, 9] and* E. granulosus* [37] TGR.

(ii) Dissociation of the products NADP ^+^ and GSH during the normal catalytic cycle (reactions 3 and 6 in Figure 11) is assumed to be irreversible steps, as revealed by the results of the product inhibition studies in the present work. However, both compounds will be able to act as competitive inhibitors of either NADPH or GSSG, respectively, through binding to their corresponding rapid equilibrium segment.

(iii) GSSG acts as a noncompetitive inhibitor by binding to the alternative site of either the reduced form of the enzyme (F) or the F-GSSG binary complex (reactions 7 and 9 in Figure 11), leading to the formation of the GSSG-F binary and the GSSG-F-GSSG ternary complexes. Binding of GSSG at the alternative site is the cause for the substrate inhibition shown in Figure 4(a). Although an alternative and simpler uncompetitive inhibition for GSSG also explains the atypical progress curves of enzyme activity of TGR, the noncompetitive inhibition pattern is more consistent with the existence of two sites. Such conclusion is supported by the statistical results of model discrimination. The potential site at which GSSG binds as inhibitor is located on the TR module at the* si *face of FAD (Figure 9(b)), near the conventional disulfide/dithiol redox center typical of the disulfide reductase family of enzymes. The affinity of the enzyme for GSSG at this alternative binding site is significantly lower (*K*i = 331 ± 79 *μ*M) compared with the affinity of the grx-like domain (*K*m = 14.4 ± 2.3 *μ*M). GSSG bound at the inhibitory site will block the electron flow from FAD to the C-terminal redox center, thus inhibiting its reduction.

(iv) It is proposed that both the GSSG-F binary and the GSSG-F-GSSG ternary complexes of the enzyme have the ability to reduce GSSG at the low-affinity alternative site (reactions 10 and 12 in Figure 11), albeit at a low rate. In such reaction the conventional N-terminal dithiol/disulfide redox center must be involved. Experimental evidence supporting this proposal is available. Thus, mutants of* S. mansoni* TGR lack either the essential selenocysteine residue or the redox active cysteines of the Grx domain [7], and also* E. granulosus* mutants in which the N-terminal cysteine residue of the Grx domain has been replaced with serine [6] are still able to catalyze the reduction of GSSG, although at a very low rate. The reported turnover numbers were 0.19 ± 0.03 s^−1^ and 0.6 ± 0.03 s^−1^ for the* S. mansoni* and* E. granulosus* enzymes, respectively. Evidence for residual GSSG reductase activity in* T. crassiceps* TGR was obtained by incubating an enzyme aliquot in the presence of a 4:1 molar excess of the irreversible inhibitor auranofin [14]. Under these conditions, a very low but measurable reductase activity was detected (turnover number 0.08 s^−1^). Hence, in the absence of either the redox active motif of the Grx-like domain or the selenocysteine residue, TGR will be still able to reduce GSSG in a GR-like fashion. The low activity of GSSG reductase at the alternative site can be explained as a result of a nonoptimal distance of the disulfide bond of GSSG after binding from the nucleophilic cysteine of the redox active motif (Figure 9(b)). In this sense, the strong dependence of the redox reactions on the distance between the electron donor and the acceptor centers is well characterized [42].

**Figure 12:**
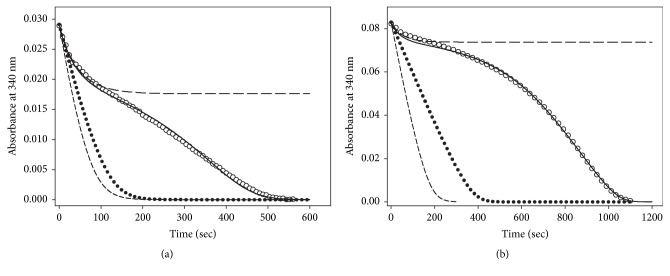
**Predictive value of the mechanism-based model.** Experimental data points (○) were compared with the full progress curves predicted by either the whole model (continuous line), the model without reactions 10-11 and 12-13 (●), or the model without reactions 11 and 13 (long dash line). The short dash line represents the profile predicted by the model in the absence of any substrate inhibition. (a) Experimental full progress curve determined at 4.7 *μ*M NADPH and 270 *μ*M GSSG. (b) Experimental full progress determined at 13.4 *μ*M NADPH and 510 *μ*M GSSG. In both cases an enzyme concentration of 11.5 nM was used. For both panels (a) and (b) the predicted full progress curves were obtained by the set of theoretical rate constants shown in Table S1 except *k*_9_ (= 0.072 s ^−1^) for panel (a) and *k*_−8_ (= 2.43 s ^−1^) and *k*_9_ (= 0.03 s ^−1^) for panel (b).

(v) As a result of the residual disulfide reductase activity of both the GSSG-F binary complex and the GSSG-F-GSSG ternary complexes, the enzyme population will be directed into the inactive states I and I-GSSG (reactions 10 and 12 in Figure 11) in which its redox centers shall be in the fully oxidized state. This proposal is in agreement with the observation that there was no glutathionylation of any catalytically essential cysteine residue when* E. granulosus* TGR was incubated with GSSG at a high concentration (1 mM) either in the presence or absence of NADPH [5]. The formation of these two fully inactive complexes explains the sudden decrease in slope in the progress curves of the reaction observed under a variety of concentrations of both NADPH and GSSG (Figure 5). By omitting reactions 10 and 12 (and hence reactions 11 and 13) from the model, the resultant full progress curves obtained by* in silico* simulations revealed conventional profiles of NADPH consumption in which GSSG acts as an reversible inhibitor but without any atypical behavior (Figure 12 and Fig.  S2).

(vi) The residual GSSG reductase activity of inhibited TGR will result in a slow but continuous increase in the concentration of the product GSH. The latter will be able to reactivate both I and I-GSSG complexes by reducing its redox active centers (reactions 11 and 13 in Figure 11) through thiol/disulfide exchange reactions, leading to a gradual increase in the concentration of the catalytically competent F and F-GSSG complexes and hence reversing the GSSG-dependent inhibition of TGR. The observation that the addition of thiol compounds such as GSH, cysteine, or DTT to the reaction mixture can abolish the hysteretic-like progress curves [5, 14, 15] strongly suggests disulfide bonds are involved in the inactivation of the enzyme by GSSG. Thus, the atypical time progress curves of GSSG reductase activity of TGR observed in the presence of moderate or high concentrations of the substrate GSSG will be the result of a continuous competition between the latter and GSH for driving the enzyme into inactive or active complexes. Such competition explains the large size that the apparent lag time can reach, extending over 1 h at high concentrations of both NADPH and GSSG. As a corollary, it can be concluded that the slope observed at the reactivating segment does not correspond to an authentic steady-state velocity. This point must be stressed, because, in some works dealing with the atypical kinetic behavior of TGR [11, 16, 37], the maximal slope in a full time progress curve of GSSG reductase activity has been mistakenly noted as a true steady-state velocity.

By deleting from the model the reactivation of the enzyme by disulfide reducing compounds (reactions 11 and 13 of Figure 11), no hysteretic-like profile of the full progress curves is obtained, as revealed by the* in silico* simulations (Figure 12 and Fig.  S3). Instead, the shape of the traces are the expected ones for an enzyme catalyzed reaction in the presence of an irreversible inhibitor [43, 44].

(vii) The effect of NADPH on the hysteretic-like full progress curves of TGR is explained as a result of providing the enzyme species (i.e., the reduced form F) to which GSSG can bind, both as substrate and as inhibitor. Hence, GSSG will behave as an uncompetitive inhibitor regarding NADPH. The unusual profile of the full progress curves observed at low concentrations of NADPH (Figure 5) but at high GSSG concentrations in which a significant burst stage is present, will be the result of the slow binding of GSSG as inhibitor to that fraction of the enzyme population in the reduced state (i.e., F and F-GSSG). It is worth noting that the best values obtained from the fitting procedure for the rate constants* k*_7_ and* k*_8_ (Table S1) suggest GSSG behave as a slow-binding inhibitor [45, 46]. Thus, in the first stages of the reaction in the presence of moderate concentrations of GSSG, the kinetic competence between the catalytic and the inhibitory reaction cycles will result in the presence of a burst-like stage.

(viii) The effect of a high concentration of GSSG on the reduction of the alternative disulfide substrate Trx by TGR further supports the proposal that the binding of GSSG occurs at the putative site located in the neighborhood of the FAD prosthetic group (Figure 9(b)). The ability of Trx to reverse the GSSG-dependent inhibition will be the result of both a competition with GSSG for the catalytically competent F form of the enzyme as well as the chemical reduction of GSSG by reduced Trx, leading to a gradual increase in GSH concentration.

The present model also can furnish an explanation of the results obtained with the* Eg*TGR C34S mutant. As reported by Bonilla et al. [6], the full time course of GR activity of such mutant in the presence of a high concentration of GSSG (1 mM) did not shows any trace of hysteretic-like behavior. By decreasing the enzyme concentration, however, the atypical profile of GSSG reductase activity was regained. Such experimental observation is explained as a result of the replacement of cysteine 34 by serine producing a more catalytically efficient enzyme (i.e., a higher turnover number). Thus, in this variant of TGR the normal catalytic cycle will be able to compete successfully with the inhibitory cycle, resulting in no observation of atypical progress curves, even at GSSG concentrations at which the inhibitory stage persists at times as long as 30 min with the wild type enzyme.

To test the predictive value of the model, simulations* in silico* of both initial velocity patterns and full progress curves were carried out. The conditions used to obtain a selected set of the specific rate constants are described in Supplementary Materials. As shown in the supplementary figures, all the experimental observations on the atypical kinetic behavior of TGR are reproduced by the model. These include the GSSG-dependent substrate inhibition (Figure S1), as well as the effect of the concentration of both GSSG and NADPH (Figures S4), GSH (Figure S6), and enzyme concentration (Figure S5), on the time progress curves. Simulations of the full progress curves using particular experimental conditions reproduce with a high consistency the experimental traces obtained under the same conditions of GSSG, NADPH and enzyme concentrations, as shown in Figures 5 and 12. Even for long time courses obtained at high concentrations of both NADPH and GSSG (Figure 5(c)), the profiles of the progress curve are consistently reproduced.

Thus, it can be concluded that the original designation of hysteretic behavior refer to the atypical full time progress curves of TGR was not correctly applied. Although apparently complex, the phenomenon is simply due to a continuous competition between a substrate (GSSG) and a product (GSH) for driving the enzyme into inactive or active states, resulting in atypical hysteretic-like progress curves. We have named this kind of kinetic behavior as pseudohysteresis. This kind of atypical progress curve of enzyme activity adds to other bizarre kinetic phenomena such as the true hysteretic behavior [21, 28] damping oscillatory hysteresis [47], and instability of the reaction product [48].

A comment concerning a recently published paper dealing with recombinant human TGR [11]. In such work, the effect of high concentrations of GSSG on enzyme activity was tested. As shown in the Figure 6 of the paper, hysteretic-like progress curves were obtained. These data further support the view that the pseudohysteretic phenomenon is common to all TGRs, independently of the presence of one or two cysteine residues at the redox active motif of the Grx-like domain.

Finally, although the substrate inhibition of TGR by moderate or high concentrations of GSSG may, in principle, appear contradictory with the function of the enzyme, it must be taken into account that the degree of inhibition is dependent on the concentration of both GSH and GSSG. As described above, 50 *μ*M GSH is high enough to avoid the GSSG-dependent inhibition of the enzyme. From the total concentration of glutathione of 1.2 mM and a GSH/GSSG ratio of 131 determined in* T. crassiceps* cysticerci under basal conditions [49] it appears that the atypical kinetic behavior of the enzyme has no physiological significance. However, taking into account the fact that under in vivo conditions the parasite is under attack by the defense system of the host, the substrate inhibition of TGR by GSSG could play a potential physiological role in the parasite survival. Thus, a sudden oxidative challenge could lead to an increase in the concentration of GSSG with a concomitant decrease in the GSH concentration, producing a temporary and partial inhibition of the enzyme activity and thus allowing the protection of essential sulfhydryl groups of proteins from oxidation through its conjugation with glutathione. In this sense, it is worth noting that the deglutathionylation activity of* E. granulosus* TGR is also inhibited by high concentrations of GSSG [6]. It is also possible that a chemical species such as the superoxide anion could participate as inhibitor of TGR, contributing to the maintenance of a relatively high, albeit temporary, concentration of GSSG.

## 5. Conclusions

- Thioredoxin-glutathione reductase from* T. crassiceps* follows a two-site ping-pong bi bi kinetic mechanism with GSSG as the substrate. The latter exerts a strong but temporary substrate inhibition, resulting in hysteretic-like progress curves.
- NADPH plays a critical role in the atypical kinetic behavior of the enzyme, by supplying the reduced form of the enzyme to which GSSG binds.
- A mechanism-based model explaining all the kinetic observations of TGR was developed. A key point of the model is the presence of a low-affinity second binding site for GSSG, with the ability to reduce GSSG at a low rate.
- From the* in silico* simulations of the model, it becomes clear that the hysteretic-like progress curves of the enzyme are the result of a continuous competition between GSH and GSSG for driving the enzyme into active or inactive pathways. Hence, the atypical full progress curves of GSSG reductase activity of TGR must not be considered as a kind of hysteretic behavior.
- As a corollary of the model, it must be stressed that the maximal slope observed in the atypical full progress curves does not represent the steady-state of enzyme activity.

## Figures and Tables

**Table 1:** Summary of kinetic parameters of *T. crassiceps* TGR.

**Parameter**	**Experimental value** ^**a**^	**Theoretical value** ^**b**^
**K**_**m**_ ** NADPH**	3.5 ± 0.4 *µ*M (n = 6)	1.8 *µ*M
**K**_**m**_ ** GSSG**	14.4 ± 2.3 *µ*M (n = 6)	6.6 *µ*M
**K**_**m**_′** GSSG**	100 ± 22 *µ*M (n = 6)	47 *µ*M
**k**_**cat**_	12 ± 2.4 s^−1^ (n = 6)	7.4 s^−1^
**k**_**cat**_/**K**_**m**_ **NADPH **	3.43 x 10^6^ s^−1^ M^−1^	4.12 x 10^6^ s^−1^ M^−1^
**k**_**cat**_/**K**_**m**_ **GSSG**	0.83 x 10^6^ s^−1^ M^−1^	1.12 x 10^6^ s^−1^ M^−1^
**K** **i** **GSSG**	331 ± 79 *µ*M (n = 6)	512 *µ*M
**K** **i** **NADP**^+^	55.3 ± 3.5 (n = 4)^c^	nd
**K** **i** **NADP**^+^	37.6 ± 5.1 (n = 4)^d^	nd

a: determined by fitting the corresponding rate equation to initial velocity data. Data represent mean ± standard deviation of the n replicates (in parenthesis); b: determined from the values of the theoretical rate constants and the definition of the corresponding kinetic parameter as derived from the mechanism-based model; c: an uncompetitive inhibition regarding GSSG; d: competitive inhibition regarding NADPH; nd not determined.

## Data Availability

The data used to support the findings of this study are available from the corresponding author upon request.

## Abbreviations

GSSG: Disulfide form of glutathione
DTNB: 5,5′-Dithiobis(2-nitrobenzoic acid)
trx: Thioredoxin
grx: Glutaredoxin
GR: Glutathione reductase
TrxR: Thioredoxin reductase
TGR: Thioredoxin-glutathione reductase.

## Supplementary Materials

Supplementary Materials
*Figure S1. In silico simulation of initial velocities of T. crassiceps TGR with GSSG as the disulfide substrate at two different NADPH concentrations*. Simulations were based on the model of Figure 11 by using the rate constants shown in Table S1. NADPH concentrations of 5 *μ*M (circles) and 50 *μ*M (triangles) were used. A value of 11.5 nM for TGR concentration was used in the simulation. Open symbols represent data obtained by omitting reactions 10 to 13 from the model.* Figure S2. In silico simulation showing the effect of omitting reactions 10 to 13 from the model on the full progress curves of T. crassiceps TGR*. Simulations were based on the model of Figure 11 by using the rate constants shown in Table S1. GSSG concentrations used were as follows: (●) 120 *μ*M; (○) 300 *μ*M; (▲) 500 *μ*M; (Δ) 800 *μ*M. NADPH and enzyme concentrations were 50 *μ*M and 11 nM, respectively.* Figure S3. In silico simulation showing the effect of omitting reactions 11 and 13 from the model on the full progress curves of T. crassiceps TGR*. Simulations were based on the model of Figure 11 by using the rate constants shown in Table S1. GSSG concentrations used were as follows: (●) 140 *μ*M; (○) 220 *μ*M; (▲) 400 *μ*M; (Δ) 550 *μ*M. NADPH and enzyme concentrations were 15 *μ*M and 11 nM, respectively.* Figure S4. In silico simulation showing the effect of varying both NADPH and GSSG concentrations on the profile of the full progress curves of T. crassiceps TGR*. Simulations were carried out at the following concentrations of NADPH: (a) 5 *μ*M; (b) 15 *μ*M; (c) 50 *μ*M. In all cases, the following concentrations of GSSG were used: (●) 60 *μ*M; (○) 120 *μ*M; (▲) 200 *μ*M; (Δ) 300 *μ*M. An enzyme concentration of 11.5 nM was used.* Figure S5. In silico simulation showing the effect of varying enzyme concentration on the profile of the full progress curves by T. crassiceps TGR*. Simulations were based on the model of Figure 11 using the rate constants shown in Table S1. (a) 7 *μ*M NADPH; (b) 50 *μ*M NADPH. In both cases the following enzyme concentrations were used: (Δ) 8.5 nM; (▲) 11.5 nM; (○) 20 nM; (●) 60 nM. A value of 350 *μ*M for GSSG concentration was used.* Figure S6. In silico simulation showing the effect of varying GSH concentration on the profile of the full progress curves by T. crassiceps* TGR. Simulations were based on the model of Figure 11 using the rate constants shown in Table S1. The following GSH concentrations were used: (Δ) none; (▲) 2 *μ*M; (○) 10 *μ*M; (●) 90 *μ*M. Values of 500 *μ*M and 11.5 nM for the concentration of GSSG and enzyme, respectively, were used.* Table S1. Best fitting theoretical rate constants used in the simulation of the model*.
