# Supplementary material for: Insight into the Mechanistic Basis of the Hysteretic-Like Kinetic Behavior of Thioredoxin-Glutathione Reductase (TGR)
Source: Enzyme Res. 2018 Sep 5;2018:3215462. doi: 10.1155/2018/3215462 (PMC6145155; doi:10.1155/2018/3215462)
Supplement: Supplementary Materials — Figure S1. In silico simulation of initial velocities of T. crassiceps TGR with GSSG as the disulfide substrate at two different NADPH concentrations. Simulations were based on the model of Figure 11 by using the rate constants shown in Table S1. NADPH concentrations of 5 μM (circles) and 50 μM (triangles) were used. A value of 11.5 nM for TGR concentration was used in the simulation. Open symbols represent data obtained by omitting reactions 10 to 13 from the model. Figure S2. In silico simulation showing the effect of omitting reactions 10 to 13 from the model on the full progress curves of T. crassiceps TGR. Simulations were based on the model of Figure 11 by using the rate constants shown in Table S1. GSSG concentrations used were as follows: (●) 120 μM; (○) 300 μM; (▲) 500 μM; (Δ) 800 μM. NADPH and enzyme concentrations were 50 μM and 11 nM, respectively. Figure S3. In silico simulation showing the effect of omitting reactions 11 and 13 from the model on the full progress curves of T. crassiceps TGR. Simulations were based on the model of Figure 11 by using the rate constants shown in Table S1. GSSG concentrations used were as follows: (●) 140 μM; (○) 220 μM; (▲) 400 μM; (Δ) 550 μM. NADPH and enzyme concentrations were 15 μM and 11 nM, respectively. Figure S4. In silico simulation showing the effect of varying both NADPH and GSSG concentrations on the profile of the full progress curves of T. crassiceps TGR. Simulations were carried out at the following concentrations of NADPH: (a) 5 μM; (b) 15 μM; (c) 50 μM. In all cases, the following concentrations of GSSG were used: (●) 60 μM; (○) 120 μM; (▲) 200 μM; (Δ) 300 μM. An enzyme concentration of 11.5 nM was used. Figure S5. In silico simulation showing the effect of varying enzyme concentration on the profile of the full progress curves by T. crassiceps TGR. Simulations were based on the model of Figure 11 using the rate constants shown in Table S1. (a) 7 μM NADPH; (b) 50 μM NADPH. In both cases the following enzy [file 3215462.f1.docx]

**Supplementary Material**

Modelling of the time progress curves.

In order to test the validity of the mechanism-based model (Figure 11) proposed to explain the atypical kinetic behavior of TGR, fitting to experimental data, and simulation of full time progress curves of NADPH consumption by the enzyme involving a non-competitive mode of inhibition by GSSG were carried out. The full model involves the following set of 13 reversible or irreversible reactions (numbered as shown in Fig. 11) with the corresponding rate constant(s):

E + NADPH <===> E-NADPH : *k* _1_ *k* _-1_ 1

E-NADPH <===> F-NADP^+^ : *k* _2_ *k* _-2_ 2

F-NADP^+^ ===> F + NADP^+^ : *k* _3_ 3

F + GSSG <===> F-GSSG : *k* _4_ *k* _-4_ 4

F-GSSG <===> E-2GSH : *k* _5_  *k* _-5_ 5

E-2GSH ===> E + 2 GSH : *k* _6_ 6

F + GSSG <===> GSSG.F` : *k* _7_ *k* _-7_ 7

GSSG-F` + GSSG <===> GSSG-F`-GSSG : *k* _4_ *k* _-4_ 8

F-GSSG + GSSG <===> GSSG-F`-GSSG : *k* _8_ *k* _-8_ 9

GSSG.F` ===> I + 2 GSH : *k* _9_ 10

I + 2 GSH ===> F + GSSG : *k* _10_ 11

GSSG.F`.GSSG ===> I-GSSG + 2 GSH : *k* _9_ 12

I.GSSG + 2 GSH ===> F.GSSG : *k* _10_ 13

The corresponding set of ordinary differential equations for the various species involved in the model is as follows:

d[E]/dt = - *k*_1_[E][NADPH] + *k*_-1_[E-NADPH] + *k*_6_[E-2GSH]

d[NADPH]/dt = - *k*_1_[E][NADPH] + *k*_-1_[E-NADPH]

d[E-NADPH]/dt = + *k*_1_[E][NADPH] – (*k*_-1_ + *k*_2_)[E-NADPH] + *k_-2_*[F-NADP]

d[F-NADP]/dt = + *k*_2_[E-NADPH] – (*k*_-2_ + *k*_3_)[F-NADP]

d[F]/dt = + *k*_3_[F-NADP] + *k_-4_*[F-GSSG] + *k*_-7_[GSSG-F] + *k*_10_[I][GSH]^2^ – (*k*_4_ + *k*_7_)[GSSG][F]

d[NADP]/dt = + *k*_3_[F-NADP]

d[GSSG]/dt = + *k*_-4_[F-GSSG] + *k*_-7_[GSSG-F] + (*k*_-4_ + *k*_-8_)[GSSG-F-GSSG] + *k*_10_[I-GSSG][GSH]^2^ +

*k*_10_[I][GSH]^2^ – *k*_4_ [GSSG-F][GSSG] – (*k*_4_ + *k*_7_)[GSSG][F] – *k*_8_[GSSG][F-GSSG]

d[F-GSSG]/dt = + *k*_4_[F][GSSG] + *k*_-5_[E-2GSH] + *k*_-8_[GSSG-F-GSSG] + *k*_10_[I-GSSG][GSH]^2^ –

(*k*_-4_ + *k*_5_) [F-GSSG] – *k*_8_[GSSG][F-GSSG]

d[GSH]/dt = + *k*_6_[E-2GSH] + *k*_9_[GSSG-F-GSSG] + *k*_9_[GSSG-F] – *k*_10_[I-GSSG][GSH]^2^ – *k*_10_[I][GSH]^2^

d[E-2GSH]/dt = + *k*_5_[F-GSSG] – (*k*_-5_ + *k*_6_)[E-2GSH]

d[GSSG-F]/dt = + *k*_7_[F][GSSG] + *k*_-4_[GSSG-F-GSSG] – *k*_4_[GSSG-F][GSSG] – (*k*_-7_ + *k*_9_)[GSSG-F]

d[GSSG-F-GSSG]/dt = + *k*_4_[GSSG-F][GSSG] + *k*_8_[GSSG][F-GSSG] – (*k*_-4_ + *k*_-8_ + *k*_9_)[GSSG-F-GSSG]

d[I]/dt = + *k*_9_[GSSG-F] – *k*_10_[I][GSH]^2^

d[I-GSSG]/dt = + *k*_9_[GSSG-F-GSSG] – *k*_10_[I-GSSG][GSH]^2^

The predicted value for all rate constants were obtained by numerical integration of the above set of differential equations through fitting to experimental progress curves. The conditions used in the fitting procedure were as follows:

i) A first set of rate constants, involving only the ping pong bi bi segment of the model (reactions 1 to 6) was obtained and then gradually refined through fitting to a variety of experimental progress curves obtained under conditions of low concentration of both NADPH and GSSG, where no atypical profile of NADPH consumption was seen. In the search for a consistent set of rate constants the following assumptions were used: a) Binding and dissociation of both substrates and products to the corresponding site on either the E or F states of the enzyme was assumed to be in rapid equilibrium; b) Dissociation of both NADP^+^ and GSH during the catalytic cycle was assumed to be irreversible, according with the kinetic evidence. Such assumption is consistent with the known irreversibility of the GSSG reduction reaction by NADPH [1]; c) Isomerization of the central complexes (E-NADPH ↔ F-NADP^+^ and F-GSSG ↔ E-2GSH) was assumed to be in steady-state; d) An initial estimate for the second order rate constant associated with the formation of the binary complexes E-NADPH and F-GSSG was obtained from the corresponding *k*_cat_/*K*_m_ ratio [2], and then gradually refined through continuous fitting.

ii) For the simulation of full time progress curves at moderate or high concentrations of GSSG, the following additional rate constants were needed: a) Reversible binding of GSSG at the inhibitory site (reactions 7 to 9). Based on the atypical profile of the full time courses this reaction was assumed as a slow one, and the corresponding rate constants were searched through fitting to a variety of experimental time courses obtained at moderate or high GSSG concentrations; b) Formation of the inactive covalent intermediaries of the enzyme (reactions 10 and 12). The initial value of the rate constant for this irreversible reaction was estimated from the initial slope of enzyme assays carried out with an auranofin-treated sample from *T. crassiceps* TGR; c) Reaction of GSH with the inactive covalent intermediaries of the enzyme through thiol/disulfide exchange reactions in order to revert the inhibition (reactions 11 and 13). The initial value of the corresponding rate constant was based on a thiol/disulfide exchange reaction involving GSH and protein disulfides [3].

The better set of rate constants obtained (Table S1) were tested for consistency with the kinetic parameters *K*_m_ (for both NADPH and GSSG), *k_cat_* and *K*_i_ as defined by the velocity equation for a ping-pong bi bi kinetic mechanism in which GSSG acts as a non-competitive inhibitor (see eq. 5 under materials and methods). The Dynafit software [4] version 4 was used for both fitting experimental data and for simulation of the full time progress curves.

[1] Y.B. Tewari, and R.N. Goldberg, Thermodynamics of the oxidation-reduction reaction

{2 glutathione _red_ (aq) + NADP _ox_ (aq) = glutathione ox (aq) + NADP red (aq)}, J. Chem.

Thermodynamics 35 (2003) 1361-1381.

[2] L. Peller, and R.A. Alberty, Multiple intermediates in steady state enzyme kinetics. I. The

mechanism involving a single substrate and product, J. Amer. Chem. Soc. (1959) 5907-

5914.

[3] H.F. Gilbert, Molecular and cellular aspects of thiol-disulfide Exchange, Adv. Enzymol. 63

(1989) 69-172.

[4] P. Kuzmic, Dynafit – A software package for enzymology, Methods Enzymol. 467 (2009)

247-280.


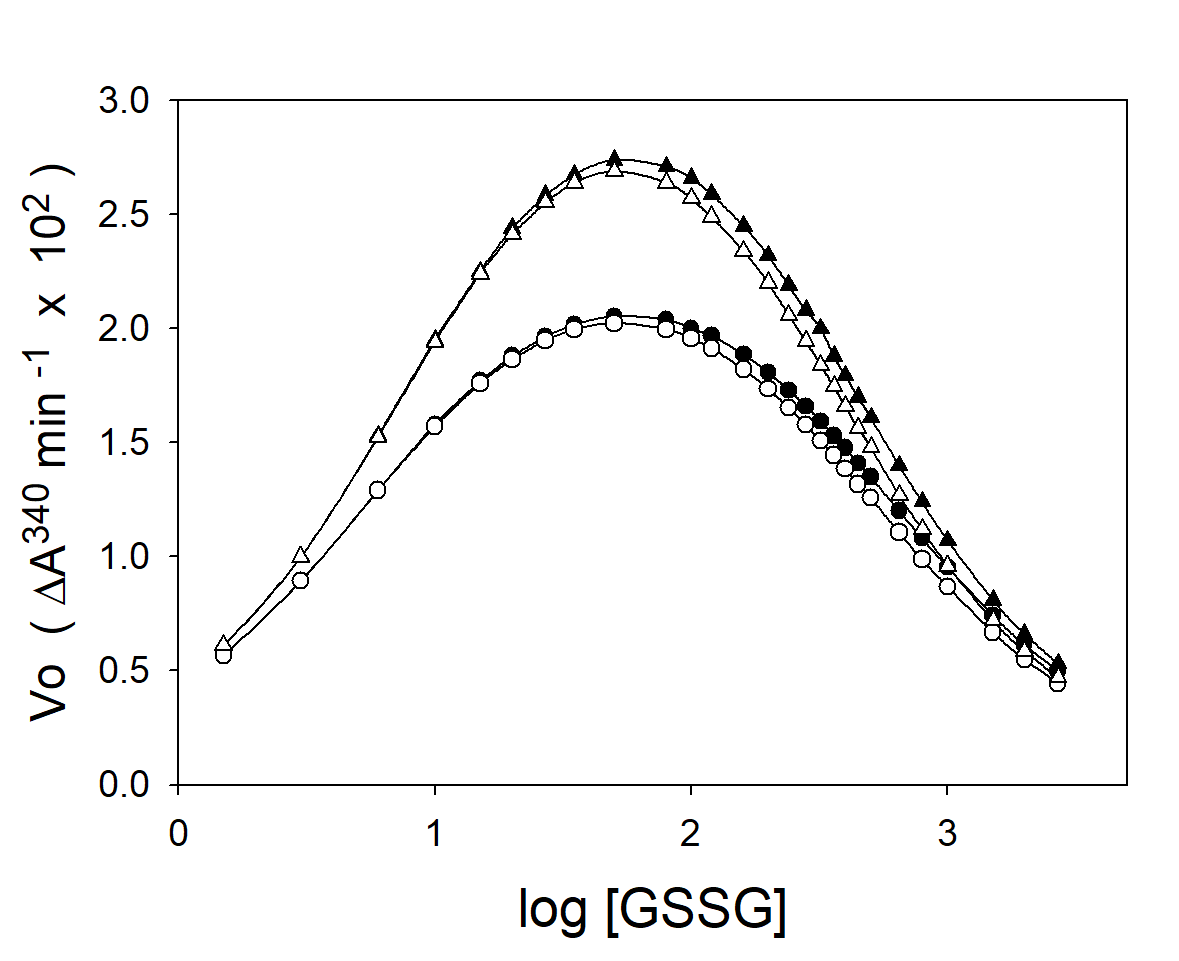


**Figure S1.** ***In silico* simulation of initial velocities of *T. crassiceps* TGR with GSSG as the disulfide substrate at two different NADPH concentrations.** Simulations were based on the model of Figure 11 by using the rate constants shown in Table S1. NADPH concentrations of 5 µM (circles) and 50 µM (triangles) were used. A value of 11.5 nM for TGR concentration was used in the simulation. Open symbols represents data obtained by omitting reactions 10 to 13 from the model.


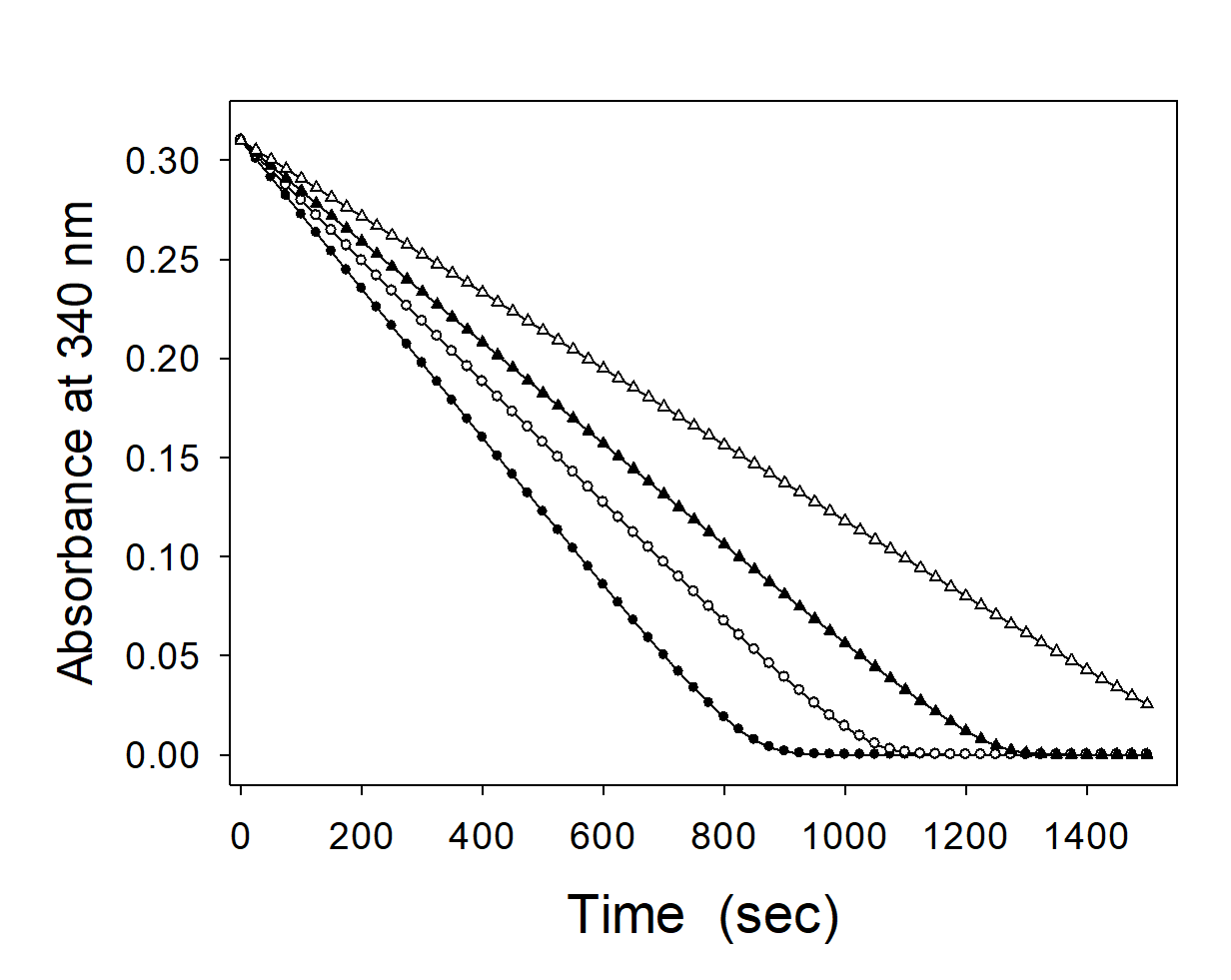


**Figure S2****. *In silico* simulation showing the effect of omitting reactions 10 to 13 from the model on the full progress curves** **of *T. crassiceps* TGR**. Simulations were based on the model of Figure 11 by using the rate constants shown in Table S1. GSSG concentrations used were as follows: (●) 120 µM; (○) 300 µM; (▲) 500 µM; (Δ) 800 µM. NADPH and enzyme concentrations were 50 µM and 11 nM, respectively.

**
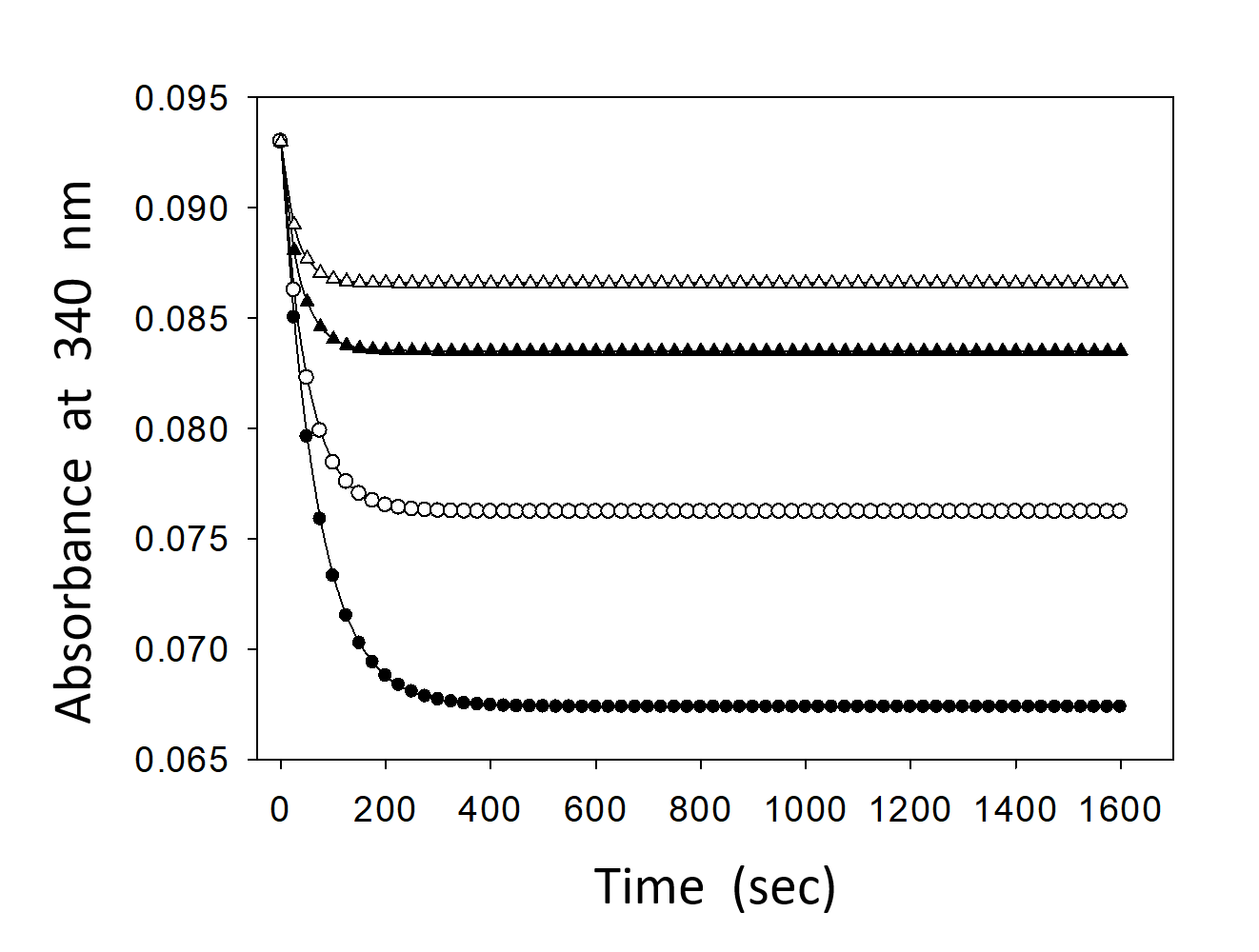
**

**Figure S3.** ***In silico* simulation showing the effect of omitting reactions 11 and 13 from the model on the full progress curves of *T. crassiceps* TGR**. Simulations were based on the model of Figure 11 by using the rate constants shown in Table S1. GSSG concentrations used were as follows: (●) 140 µM; (○) 220 µM; (▲) 400 µM; (Δ) 550 µM. NADPH and enzyme concentrations were 15 µM and 11 nM, respectively.


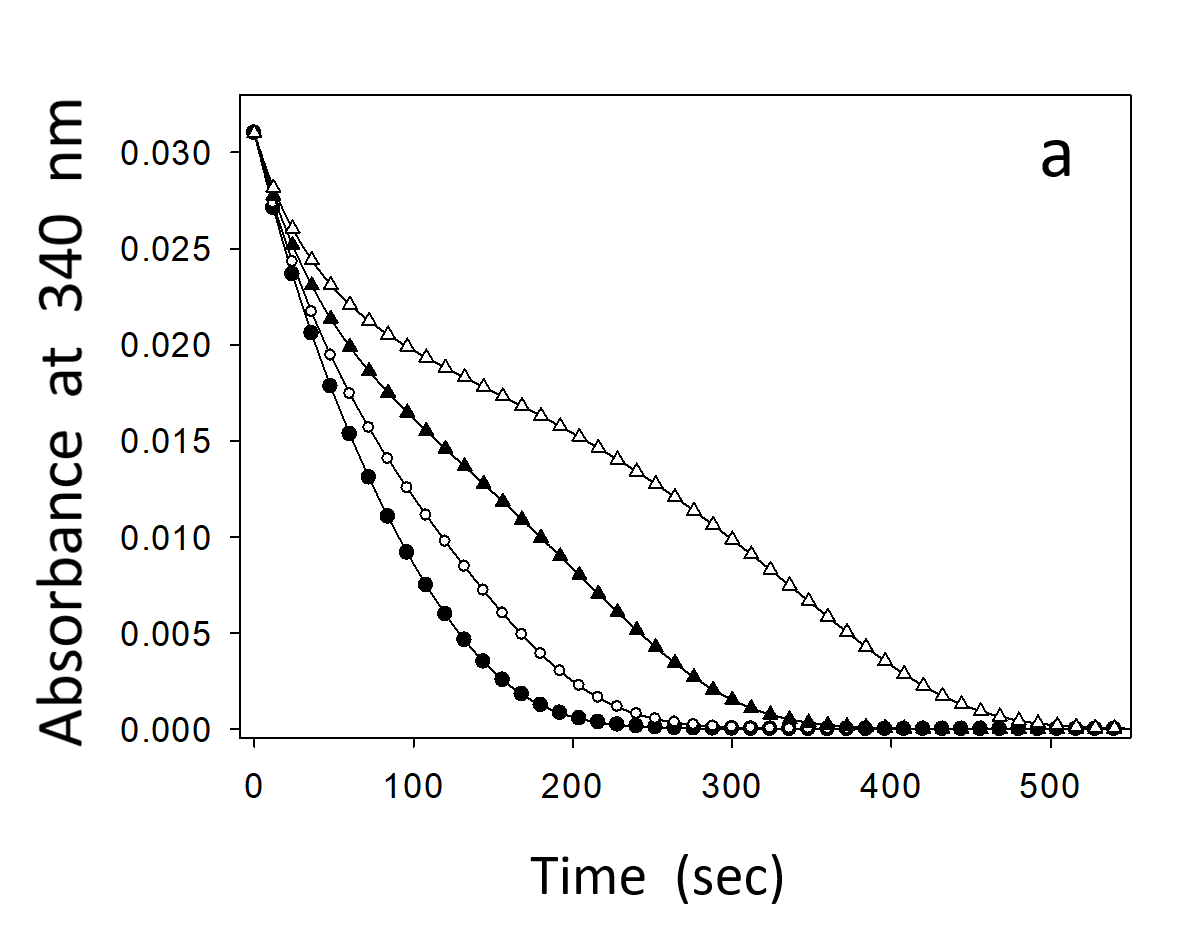


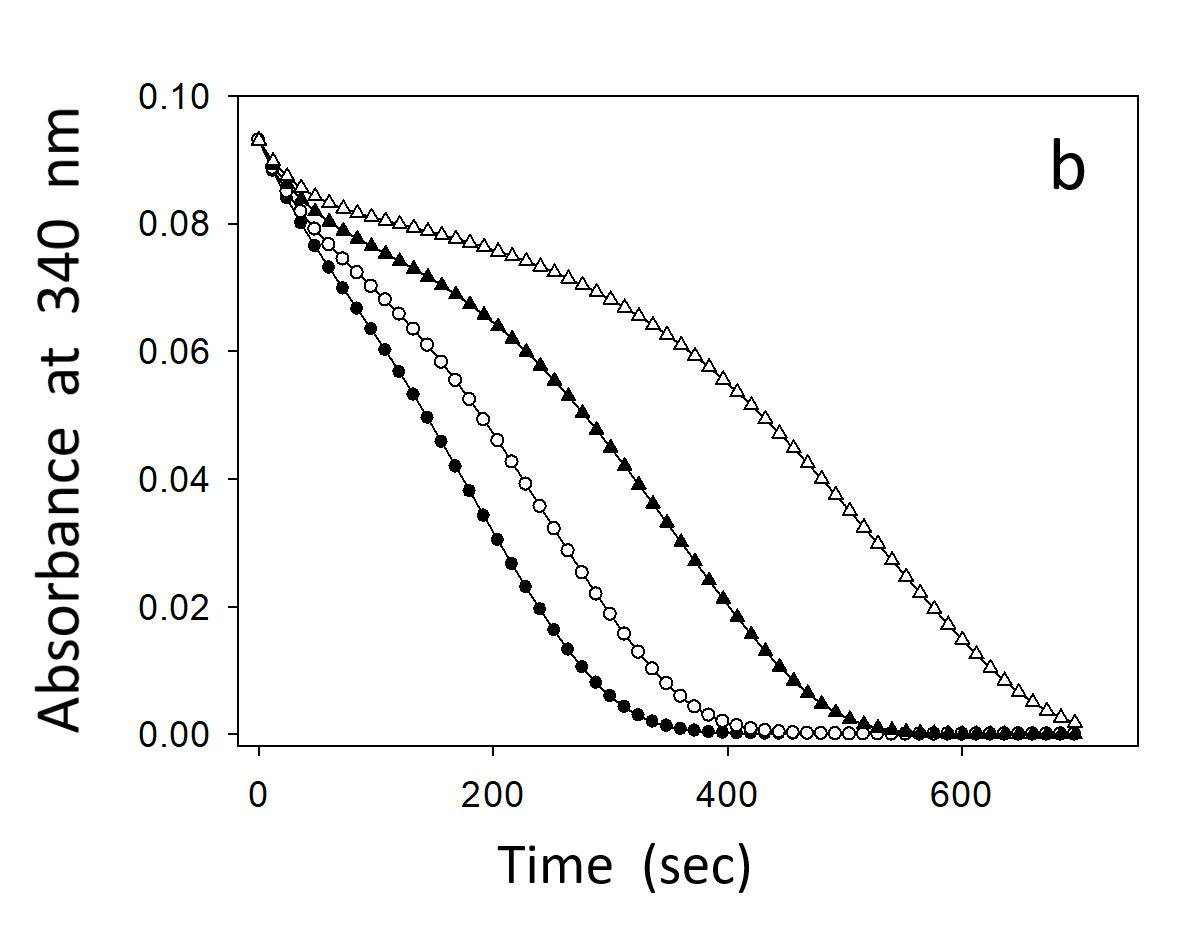


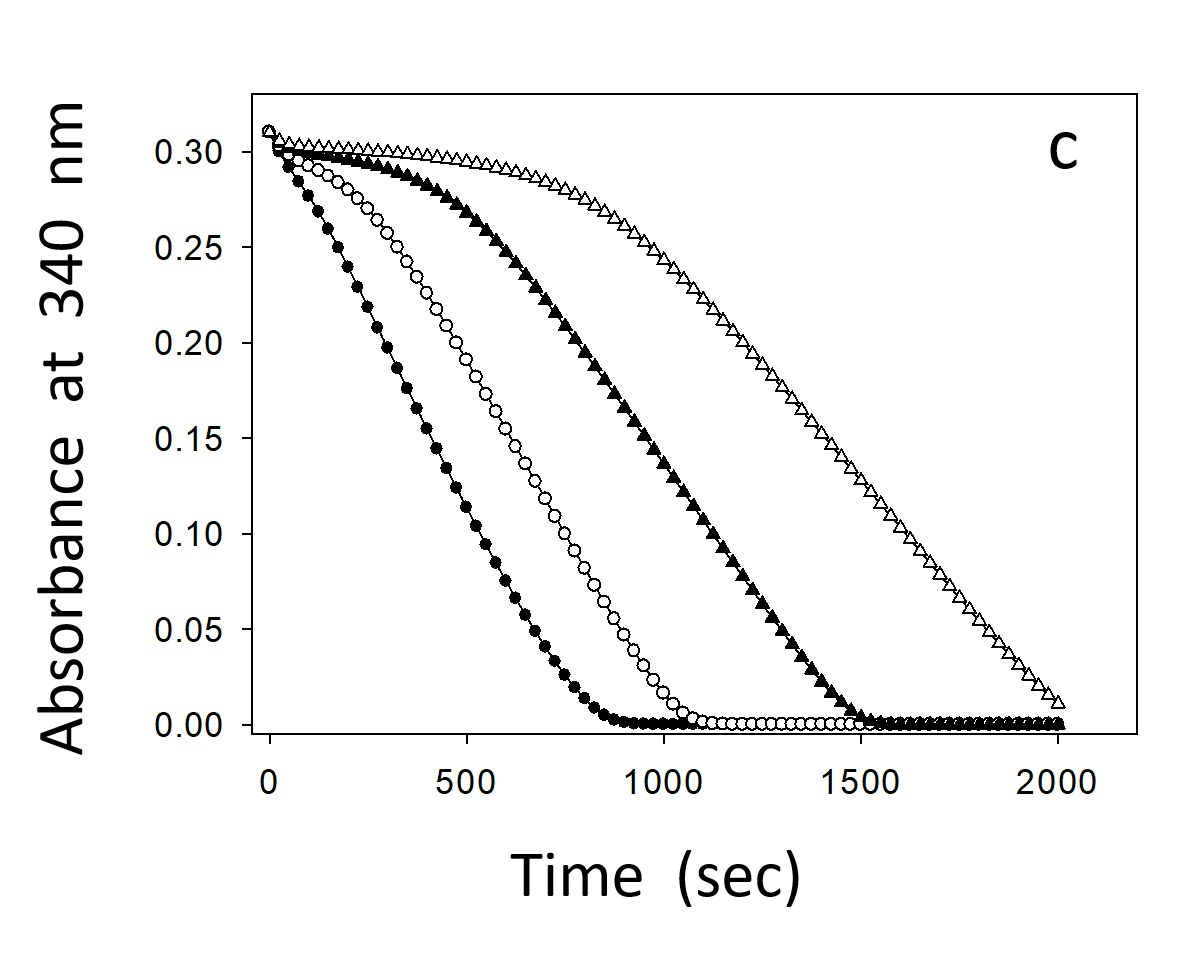


**Figure S4. *In silico* simulation showing the effect of varying both NADPH and GSSG concentrations on the profile of the full progress curves of *T. crassiceps* TGR**. Simulations were carried out at the following concentrations of NADPH: a) 5 µM; b) 15 µM; c) 50 µM. In all cases, the following concentrations of GSSG were used: (●) 60 µM; (○) 120 µM; (▲) 200 µM; (Δ) 300 µM. An enzyme concentration of 11.5 nM was used.


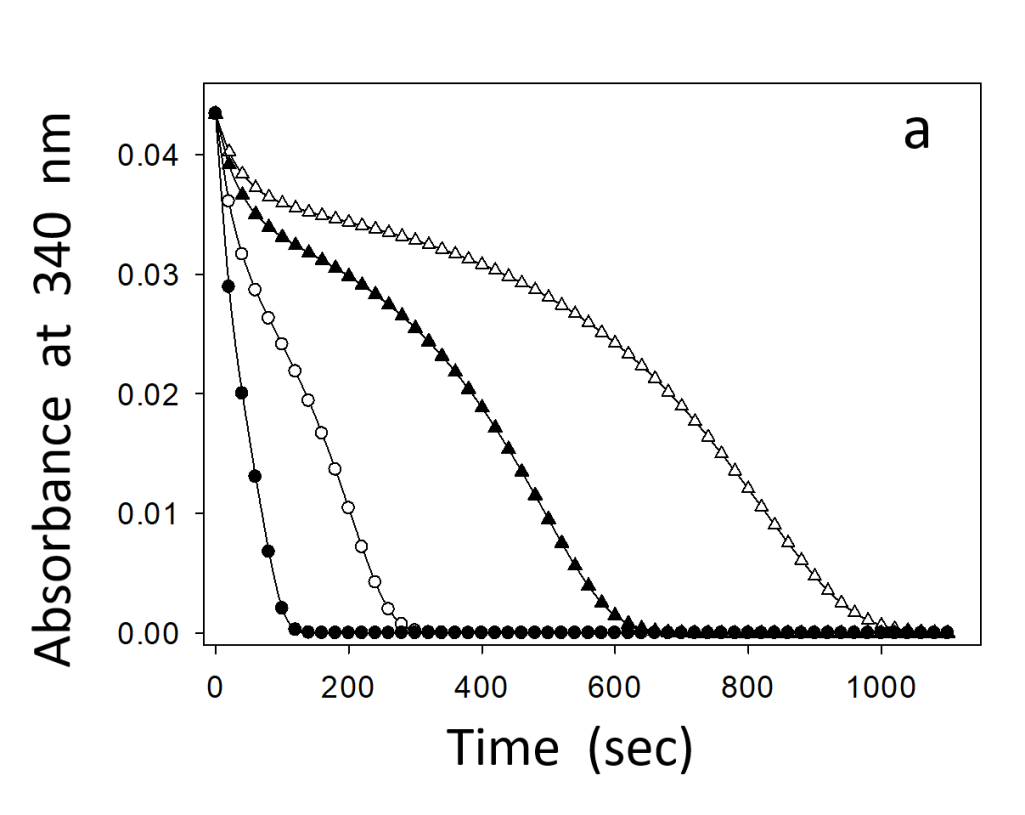


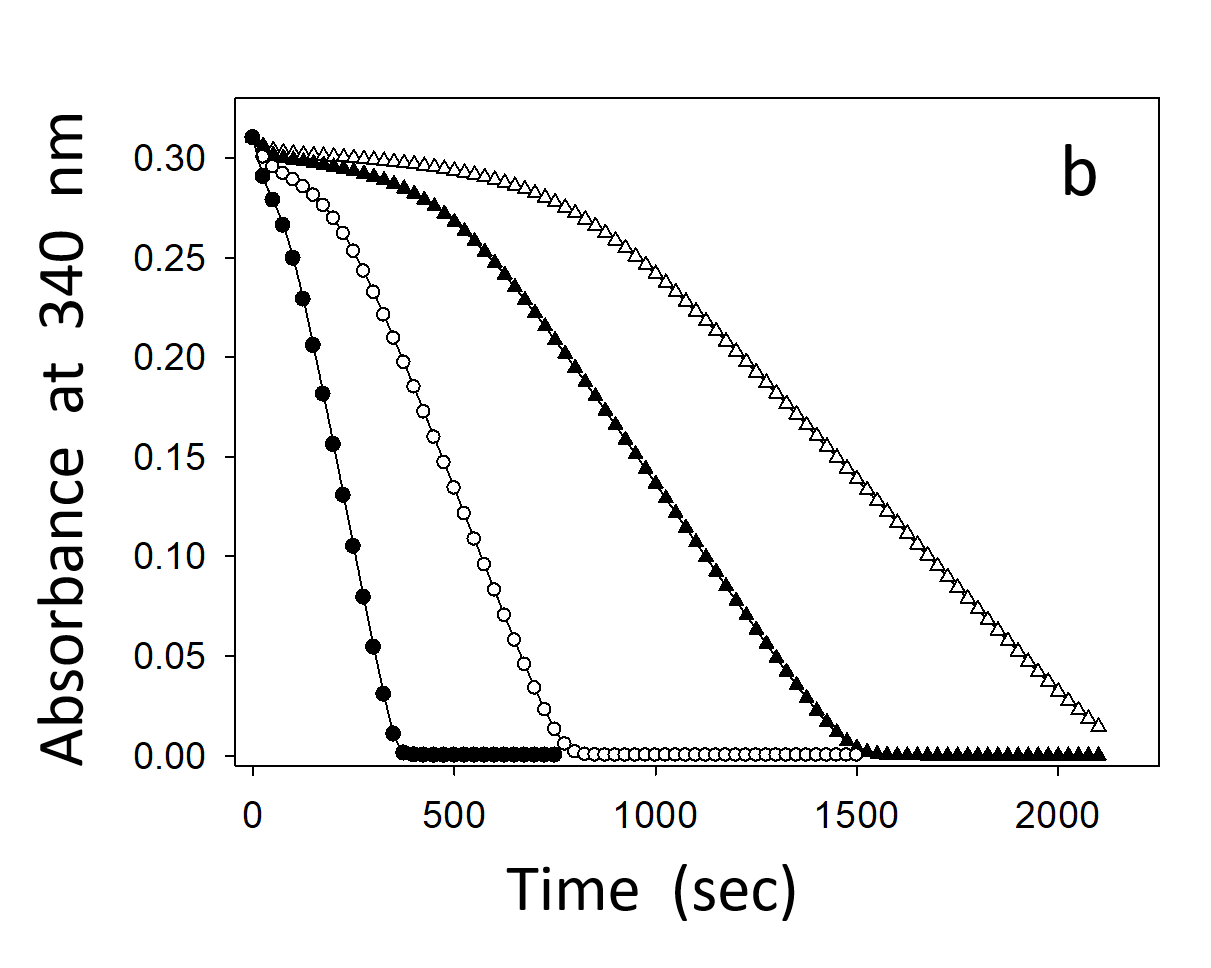


**Figure S5.** ***In silico* simulation showing the effect of varying enzyme concentration on the profile of the full progress curves by *T. crassiceps* TGR**. Simulations were based on the model of Figure 11 using the rate constants shown in Table S1. a) 7 µM NADPH; b) 50 µM NADPH. In both cases the following enzyme concentrations were used: (Δ) 8.5 nM; (▲) 11.5 nM; (○) 20 nM; (●) 60 nM. A value of 350 µM for GSSG concentration was used.


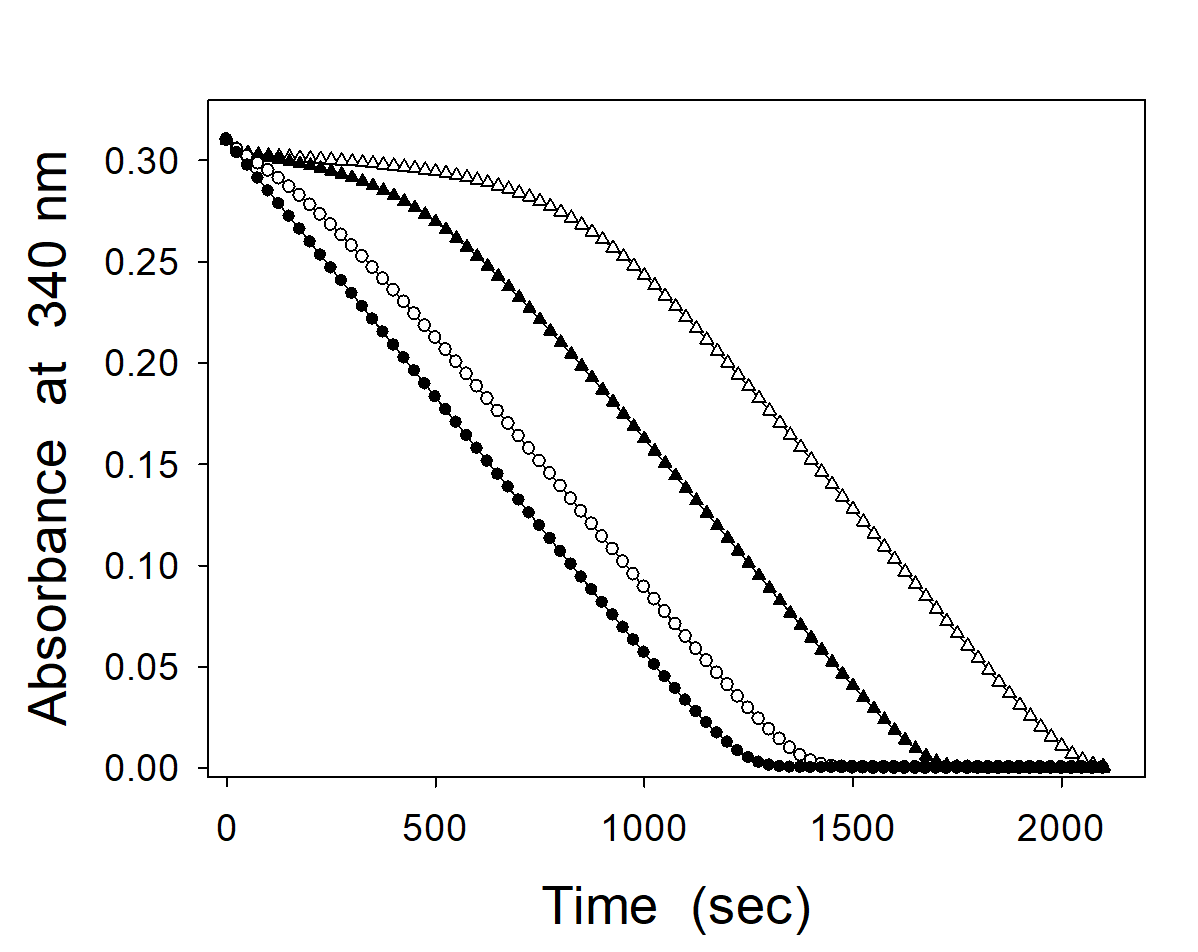


**Figure S6.** ***In silico* simulation showing the effect of varying GSH concentration on the profile of the full progress curves by *T. crassiceps*** TGR. Simulations were based on the model of Figure 11 using the rate constants shown in Table S1. The following GSH concentrations were used: (Δ) none; ( ▲ ) 2 µM; ( ○ ) 10 µM; ( ● ) 90 µM. Values of 500 µM and 11.5 nM for the concentration of GSSG and enzyme, respectively, were used.

| Ping Pong Segment | Best Fit Value  (theoretical) |
| --- | --- |
| *k* _1_ | 25 µM ^-1^ s ^-1^ |
| *k* _-1_ | 480 s ^-1^ |
| *k* _2_ | 90 s ^-1^ |
| *k* _-2_ | 20 s ^-1^ |
| *k* _3_ | 160 s ^-1^ |
| *k* _4_ | 12.5 µM ^-1^ s ^-1^ |
| *k* _-4_ | 171 s ^-1^ |
| *k* _5_ | 26 s ^-1^ |
| *k* _-5_ | 22 s ^-1^ |
| *k* _6_ | 32 s ^-1^ |
| Substrate Inhibition Segment |  |
| *k* _7_ | 0.075 µM ^-1^ s ^-1^ |
| *k* _-7_ | 17 s ^-1^ |
| *k* _8_ | 0.02 s ^-1^ |
| *k* _-8_ | 4.2 s ^-1^ |
| Reactivating Segment |  |
| *k* _9_ | 0.06 s ^-1^ |
| *k* _10_ | 2.3 x 10 ^-4^ µM ^-1^ s ^-1^ |

**Table S1. Best fitting theoretical rate constants used in the simulation of the model**
